# Complete genomes reveal a refined map of *Mycobacterium tuberculosis* genetic diversity across evolutionary scales

**DOI:** 10.1038/s41467-026-73869-5

**Published:** 2026-06-06

**Authors:** Ana María García-Marín, Manuela Torres-Puente, Llúcia Martinez-Priego, Griselda De Marco, Miguel Moreno-Molina, Martin Hunt, Zamin Iqbal, Ana Gil-Brusola, Valencia Region TB Working Group, Ana Gil-Brusola, Aurora Blasco, Bárbara Gomila-Sard, José Luis López-Hontangas, Rafael Borrás, María Angeles Clari, Javier Colomina, David Navarro, Concepción Gimeno, María Remedio Guna-Serrano, Juan J. Camarena, Ester Colomer-Roig, Nieves Orta, María M. Ruiz-García, Nieves Gonzalo-Jiménez, Adelina Gimeno-Gascón, Juan Carlos Rodríguez, María Borrás-Máñez, Isabel Escribano, Olalla Martinez-Macias, Oscar Esparcia-Rodríguez, Mariana G. López, Iñaki Comas, Mariana G. López, Fernando González-Candelas, Javier Alonso-del-Real, Iñaki Comas

**Affiliations:** 1 Tuberculosis Genomics Unit, Instituto de Biomedicina de Valencia (IBV-CSIC), Valencia, Spain; 2 Joint Research Unit ‘Infección y Salud Pública’ FISABIO, University of Valencia-I2SysBio, Valencia, Spain; 3 Sequencing and Bioinformatics Genomic Core Unit, FISABIO-Salut Pública, València, Spain; 4 European Bioinformatics Institute, Hinxton, UK; 5 Nuffield Department of Medicine, University of Oxford, Oxford, UK; 6 National Institute of Health Research Oxford Biomedical Research Centre, John Radcliffe Hospital, Headley Way, Oxford, UK; 7 Health Protection Research Unit in Healthcare Associated Infections and Antimicrobial Resistance, University of Oxford, Oxford, UK; 8 Milner Centre for Evolution, University of Bath, Bath, UK; 9 Microbiology Service, Hospital Universitari i Politècnic La Fe, Valencia, Spain; 10 CIBER of Epidemiology and Public Health (CIBERESP), Madrid, Spain; 11 Microbiology Service, Hospital General Universitario de Castellón, Castellón, Spain; 12 Microbiology Service, Hospital Clínico Universitario, Valencia, Spain; 13 Departamento de Microbiología. Facultad de Medicina. Universidad de Valencia, Valencia, Spain; 14 Microbiology Service, Consorcio Hospital General Universitario de Valencia, Valencia, Spain; 15 Microbiology Service, Hospital Universitario Dr. Peset, Valencia, Spain; 16 Microbiology Service, Hospital Francesc de Borja, Gandía, Spain; 17 Microbiology Service, Hospital General Universitario de Elche, Elche, Spain; 18 CIBERINFEC, ISCIII, Madrid, Spain; 19 Microbiology Service, Hospital General Universitario de Alicante, Alicante, Spain; 20 Microbiology and Parasitology Service, Hospital Universitario de La Ribera, Alzira, Spain; 21 Microbiology Laboratory, Hospital Virgen de los Lírios, Alcoy, Spain; 22 Microbiology Service, Hospital de Denia, Denia, Spain

**Keywords:** Bacterial genetics, Genome informatics, Tuberculosis, Evolutionary genetics, Genetic variation

## Abstract

Elucidating the evolution and epidemiology of *Mycobacterium tuberculosis* requires comprehensive characterization of its genomic diversity; however, short-read sequencing fails to resolve part of this variation. Here, we assembled 216 complete genomes from clinical isolates in the Valencia Region, Spain, using long-read sequencing. This dataset, mostly encompassing Lineage 4, provides a refined map of *M. tuberculosis* genetic diversity across evolutionary scales. Complete genomes uncover a median of 312 (−1 to 792) additional SNPs per pairwise comparison, revealing an estimated evolutionary rate 1.44-fold higher than that inferred from short-read mapping. This diversity is concentrated in discrete hotspots, particularly within the *pe/ppe* gene family, where gene conversion is a major driver of nucleotide diversity. While most PE/PPE epitopes remain highly conserved, suggesting strong purifying selection, some involved in vaccine candidates are affected by gene conversion, with unknown consequences. At the epidemiological scale, additional resolution is gained from SNPs previously masked and newly resolved indels and structural variation, refining genetic transmission networks. Finally, at the within-host level, the use of patient-specific reference genomes allows us to capture genuine diversity during infection, showing that previous approaches led to false positive calls. Together, these findings delineate the landscape of *M. tuberculosis* genomic diversity and provide a framework for more accurate inference of pathogen evolution, host–pathogen interactions, and transmission dynamics.

## Abstract (web summary)

García-Marín et al. reveal hidden genetic diversity in *Mycobacterium tuberculosis* by assembling 216 complete genomes from long-read sequencing data. The work provides a framework for more accurate inference of pathogen evolution, host–pathogen interactions, and transmission dynamics.

## Introduction

Despite advances in diagnosis, surveillance, and treatments, tuberculosis (TB) remains a major global health problem^1^. The widespread use of short-read sequencing has enabled the rapid and large-scale sequencing of the *Mycobacterium tuberculosis* complex (MTBC), providing new insights into its genetic diversity. This has fueled extensive efforts to link MTBC genetic variation to key phenotypes such as virulence, transmission, drug resistance, host tropism, and disease manifestations^2^.

However, genomic studies are hampered by the limitations of short-read sequencing in resolving repetitive or highly complex regions, which are excluded from bioinformatic analyses due to mapping challenges^3^. As a consequence, approximately 5-10% of the genome cannot be accurately assessed and remains understudied^4,5^. Paradoxically, some of the most diverse and functionally relevant regions of the genome, such as *pe/ppe* genes, are inaccessible by short-read sequencing^4,6,7^. Those regions also harbor the potential to increase the resolution of genomic transmission studies, which also overlook other sources of variation beyond point mutations. We are therefore blinded to regions that can potentially improve our understanding of tuberculosis disease and epidemiology.

Another common limitation of reference-mapping approaches is the use of a single reference genome to analyse strains across all *Mycobacterium tuberculosis* (MTB) lineages, typically H37Rv or an H37Rv ancestral-like sequence that preserves genome architecture while incorporating ancestral alleles^8^. Although MTB is often described as monomorphic, reference choice can influence genetic diversity estimates^9–11^. This issue is particularly relevant for the detection of within-host variation, where non-fixed variants are prone to false-positive calls, challenging reliable inference^12^. Accurate identification of within-host variation is critical, as it can reveal heteroresistance and selective processes during host–pathogen interactions, and can be used to increase the resolution of genomic transmission networks, as shown by Lee et al.^13^.

Long-read sequencing technologies are currently the only viable alternative to overcome these limitations since they allow for the accurate reconstruction of the complete MTB genome through de novo assembly^3^. Previous studies using MTB complete genomes were constrained by small sample sizes and the moderate quality of long-read data, often requiring polishing with short reads or hybrid assembly approaches^14–16^. Still, progress has been made to address specific questions, although sample sizes preclude the generalization of results in many cases^17–19^. Finally, while the accuracy of long-read sequencing has improved over the last few years^20^, sequencing results are still limited by the low quality of bacterial DNA (resulting from harsh conditions of extraction protocols) and the high cost of these technologies^20^.

Here, we present the largest dataset of high-quality complete MTB genomes with matched short-read sequences published to date, comprising 216 MTBC clinical isolates collected in 2016 from the Valencia Region, Spain. We generated complete genome sequences solely from long-read HiFi sequencing data, achieving exceptional quality without including short reads for polishing or performing hybrid approaches.

The global analysis of this dataset, enriched in Lineage 4, reveals that complete genomes capture substantially more genetic diversity than the matched short-read data. Most genetic diversity hotspots map to the *pe/ppe* gene family, a largely uncharacterized group in mycobacteria, with gene conversion emerging as the main driver of nucleotide diversity in these regions. Despite the high overall variability of *pe/ppe* genes, epitopes from PE/PPE antigens are hyperconversed, with notable exceptions included in vaccine candidates. At the epidemiological scale, incorporating SNPs from overlooked regions, indels, and structural variants provides higher resolution for defining genetic transmission boundaries and networks. Finally, we use a complete genome from the same patient as a reference to map short reads from serial isolates to reliably detect genuine within-host variation. This strategy reveals a substantial reduction in non-fixed variation due to false positives introduced by conventional pipelines using a global reference, prompting a reassessment of current within-host diversity estimates and the dynamics of *M. tuberculosis* during infection.

## Results

### A unique dataset of paired high-quality MTBC complete genomes and short-read data

Of 266 MTBC culture-positive isolates available in the Valencia Region of Spain from 2016, 216 (80%) were sequenced using the PacBio HiFi method. An average sequencing depth of 100.5x was achieved with a mean read length of 4505 (see Supplementary Note 1, Supplementary Fig. 1, and Supplementary Data 1 for more statistics). We achieved 212 genome assemblies closed in a single circular contig, and all genome assemblies were highly accurate and complete. A detailed description of parameters used to assess contiguity, completeness, and correctness can be found in Table 1, Supplementary Note 1, and Supplementary Data 2. As the 216 assemblies demonstrated consistently high-quality metrics, they were all included in downstream analyses.

**Table 1:** Quality control parameters of genome assemblies classified by the 3 C criteria

	Parameter	Median (range)
**Contiguity**	Number of contigs	1 (1–7)
	Genome size	4,383,653 (4,339,847–4,452,994)
	Circularization	212 yes, 4 no
	N50	4,408,709 (220,246–4,452,994)
**Correctness**	Short-read k-mer based completeness	99.95% (99.13–99.99%)
	QV	42.59 (36.84–58.53)
	Short-read mismatches	0 (0–3)
	Percentage of properly mapped short reads	99.96% (99.74–99.99%)
	Horizontal coverage of short reads	99.76% (99.36–99.99%)
**Completeness**	Complete BUSCOs	99.4% (99.0–99.6%)
	Difference in mean length of predicted proteins compared to H37Rv	1 (−1 to 3) amino acid
	Difference in % of predicted proteins with length >95% compared to H37Rv	0.04% (−1.23% to −0.43%)
	Misassembly	0 (0–0)

Table 1 summarizes parameters for assembly quality control. They are grouped according to the 3 C criteria: contiguity, completeness, and correctness; some of them can be representative of more than one category. Most of the metrics were outstanding for all 216 genome assemblies.

Short-read data were available for all 216 MTBC isolates, with a mean coverage depth of 130x. The lineage (L) distribution of the MTBC samples was as follows: 4 (2.0%) from L2, 2 (0.9%) from L3, and 209 (96.7%) from L4. In addition, one sample (0.4%) was identified as *M. bovis*. 194 (89.8%) isolates were susceptible to all first-line antituberculous drugs, and 22 (10.2%) isolates were resistant to at least one antibiotic (drug resistance profiles are detailed in Supplementary Fig. 1 and Supplementary Data 1).

### Complete genomes reveal greater genetic diversity and a higher evolutionary rate

**Fig. 1:**
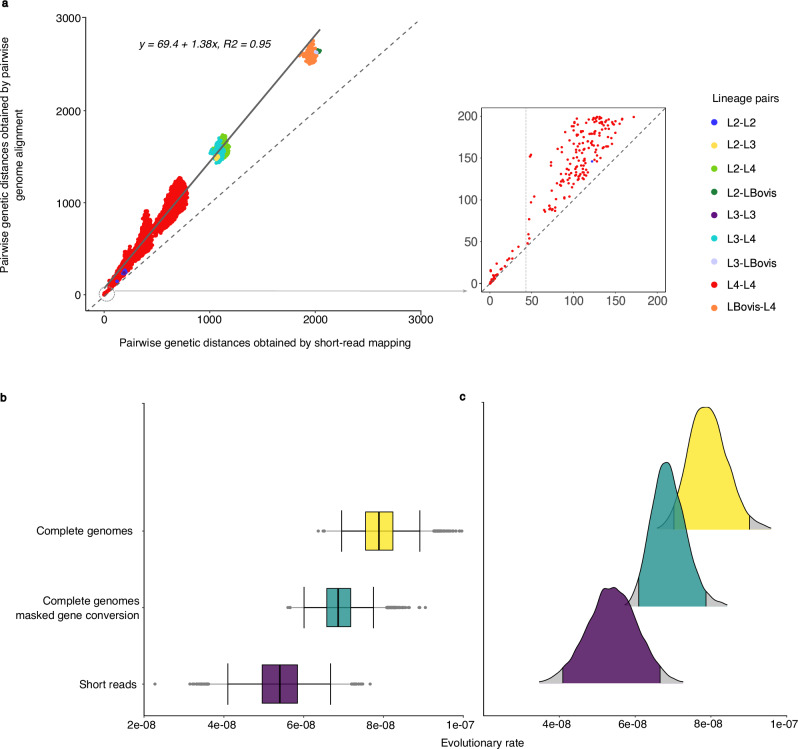
Comparison of pairwise genetic distances and evolutionary rates using short-read and complete genome data. **a** Comparison of pairwise genetic distances between MTBC isolates using short-read and complete genome data. Each point represents a pairwise comparison between the 216 MTBC isolates, with SNP distances obtained from short-read data on the x-axis and complete genomes on the y-axis. The dashed gray line represents the identity line (x = y), while the solid line indicates the linear regression. Notably, almost all comparisons fall above the identity line, indicating higher genetic distances obtained from complete genomes. Colors indicate the lineages of the strains involved in each pairwise comparison. The three differentiated dot clusters correspond to varying levels of divergence: the first includes comparisons between isolates of the same lineage, the second between human-adapted lineages, and the last between *M. bovis* and human-adapted lineages. This illustrates how the number of SNPs gained increased with the level of divergence between the isolates, thus, when comparing isolates from distinct lineages. The inset zooms in on the region with ≤200 SNPs in both approaches, and the vertical dashed line marks the genetic distance obtained by short reads at which comparisons begin to scatter. It shows that pairs of isolates with closer genetic distance (based on short-read data) showed a substantially lower SNP count discrepancy that increased sharply when comparing more genetically distant isolates. Comparison of evolutionary rates estimated with: short-read data (purple), complete genomes (yellow), and complete genomes masking gene conversion in *pe/ppe* genes (blue) for 209 MTB lineage 4 isolates from this dataset. The number of substitutions/site/year is indicated on the x-axis. For each replicate, two distributions are shown: a boxplot (**b**) and a density plot (**c**). In the box plots, the center line represents the median, and the box bounds represent the first and third quartiles (the interquartile range, IQR). The whiskers extend to the smallest and largest values (minima and maxima) that are within 1.5 times the IQR from the box bounds. Data points beyond this range are plotted individually as outliers. Source data are provided as a Source Data file.

To assess the performance of the complete genome approach in detecting SNPs, we measured the pairwise genetic distances between each pair of samples comparing complete genomes from the MGA (see Supplementary Note 2) and short-read data. The two measures were strongly correlated (Spearman’s rho = 0.87, *p* < 2.2 × 10^−16^, *n* = 23220), with complete genomes consistently yielding equal or greater pairwise genetic distances than those obtained by short-read mapping, showing an average of 303 and a median of 312 (−1 to 792) additional SNPs per pair (Fig. 1a). Of the additional SNPs identified, 268/303 (88%) were in regions discarded by the short-read mapping approach, while 35/303 (12%) were found in preserved regions. Moreover, the phylogeny of complete genomes was not significantly different than the short-read derived (SH test *p* ≈ 1), with a strong correlation between all branch lengths (Spearman’s rho = 0.98, *p* < 2.2 × 10–16, *n* = 430). These results suggest that the additional variation was evenly distributed throughout the phylogeny, with no evidence of accumulation in specific branches.

Additionally, to quantify the additional variation, we estimated evolutionary rates for *M. tuberculosis* lineage 4 strains using both short reads and complete genomes (Fig. 1b, c). For the short-read sequences, the estimated median evolutionary rate was 5.40 × 10⁻⁸ substitutions/site/year, with a 95% highest posterior density (HPD) interval of 4.10 × 10⁻⁸ to 6.67 × 10⁻⁸. In contrast, the complete genome analyses yielded consistently higher rates across three prior scenarios: i) 7.84 × 10⁻⁸ [95% HPD: 6.47 × 10⁻⁸ – 9.50 × 10⁻⁸]; ii) 7.88 × 10⁻⁸ [6.96 × 10⁻⁸ – 8.93 × 10⁻⁸]; and iii) 7.60 × 10⁻⁸ [7.31 × 10⁻⁸ – 7.88 × 10⁻⁸] (Supplementary Fig. 2). Overall, evolutionary rate estimates derived from complete genome data were 1.44-fold higher than those from short reads. After masking the gene conversion events, we obtained the following evolutionary rates across the same three prior scenarios: i) 6.84 × 10⁻⁸ [95% HPD: 5.69 × 10⁻⁸ – 8.29 × 10⁻⁸]; ii) 6.86 × 10⁻⁸ [6.01 × 10⁻⁸ – 7.75 × 10⁻⁸]; and iii) 6.62 × 10⁻⁸ [6.37 × 10⁻⁸ – 6.88 × 10⁻⁸] (Supplementary Fig. 2). In this case, the evolutionary rate was 1.25-fold higher than that from short reads.

### Global-scale analysis of complete genomes unveils genetic diversity hotspots across the MTB genome

**Fig. 2:**
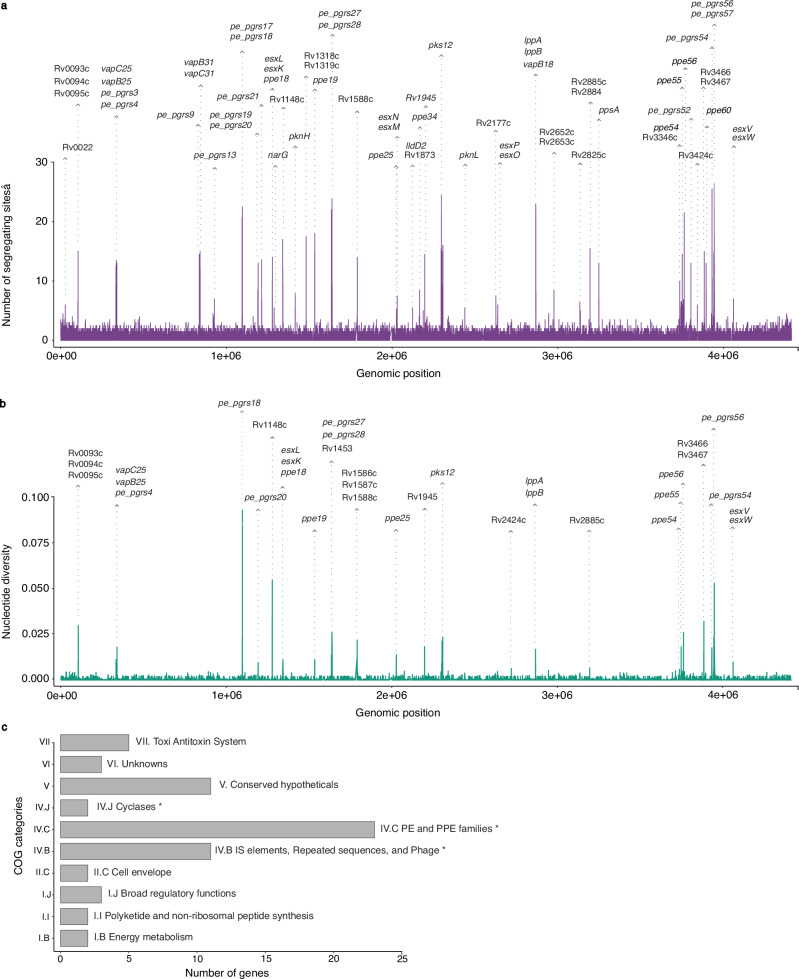
Genomic diversity of *Mycobacterium tuberculosis* complex. Sliding window analysis showing (**a**) the number of segregating sites and (**b**) the nucleotide diversity in blue across the refined MGA with 216 MTBC complete genomes. The window length was 200 bp with a step size of 1 bp. The x-axis indicates the position of the windows’ midpoint. **c** Distribution of COG categories of genes with the highest levels of segregating sites. The x-axis shows the number of genes per category, and the COGs are displayed on the y-axis. The functional analysis identified the PE and PPE families as major contributors to diversity within our dataset (see also Supplementary Table 1); *, adjusted *p* < 0.01. Source data are provided as a Source Data file.

From the pairwise genetic distance analysis, we found that 4804/25865 (20%) polymorphic sites were in regions not assessed by standard short-read approaches (see Supplementary Note 2). To characterize the total MTB diversity in our dataset, including those regions, we performed a sliding window analysis across the refined MGA (Supplementary Note 2). Segregating sites between complete genomes and the MTBCA reference genome were analyzed to assess genomic divergence since the emergence of a common ancestor. This analysis identified 94 high-diversity genomic regions, including 64 genes and 30 intergenic regions (Fig. 2a, Supplementary Data 3). Based on the masking filter applied in the short-read mapping approach, 55/94 (60%) were in masked segments, while 38/94 (40%) were in preserved segments. Additionally, nucleotide diversity was measured between strains in our population dataset, considering that most isolates belonged to Lineage 4. The analysis identified 52 high-diversity hotspots; 40/52 (77%) were in discarded and 12/52 (23%) in preserved regions (Fig. 2b, Supplementary Data 3).

Genes with diversity hotspots identified by segregating sites were distributed across 10 MTB COG (Clusters of Orthologous Groups) categories. The functional enrichment analysis revealed significant overrepresentation in the following groups: IV.B IS elements, repeated sequences, and phages (adjusted *p* = 6.86 × 10^−5^), IV.J Cyclases (adjusted *p* = 0.02), and IV.C PE and PPE families (adjusted *p* = 2.58 × 10^−14^) (see Fig. 2c and Supplementary Table 1). All statistical analyses were performed using Fisher’s Exact Test, and adjusted *p*-values were obtained by applying the Benjamini-Hochberg correction.

### Gene conversion is a main source of nucleotide diversity in the *pe/ppe* gene family

**Fig. 3:**
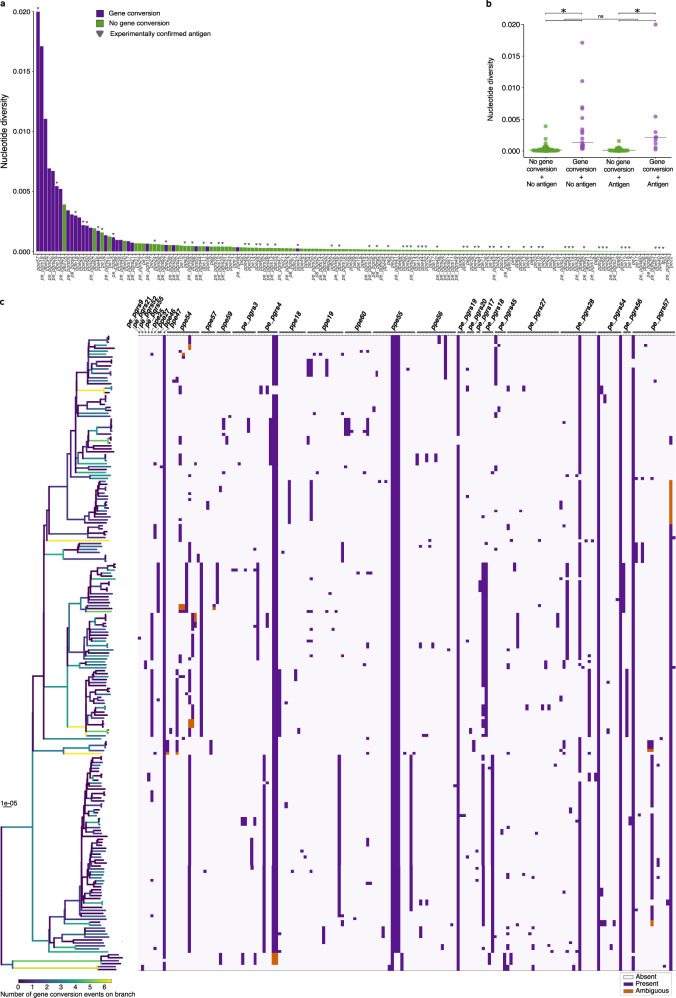
Genetic diversity of the *pe/ppe* gene family. **a** Distribution of nucleotide diversity of 169 *pe/ppe* genes. Genes with evidence of gene conversion events are in red, while the ones without evidence of gene conversion are in blue. Genes that encode a PE/PPE antigen are marked with a grey triangle. **b** Distributions of nucleotide diversity of four *pe/ppe* gene categories. *Pe/ppe* genes were classified based on the presence of gene conversion events and whether they encode antigens: genes with gene conversion that encode antigen (*n* = 9), genes with gene conversion that do not encode antigens (*n* = 19), genes without gene conversion that encode antigen (*n* = 52), and genes without gene conversion that do not encode antigen (*n* = 89). The distribution of nucleotide diversity was plotted for each category. Brackets indicate two-sided Wilcoxon Rank-Sum Tests of pairwise category comparisons; ns, not significant; *p < 0.01. Specific *p*-values for each comparison are as follows: non-antigen-encoding genes with vs. without gene conversion (*p* = 9.1 × 10^-10^); antigen-encoding genes with vs. without gene conversion (*p* = 1.1 × 10^-5^); and non-antigen-encoding vs. antigen-encoding genes (*p* = 0.15). **c** Phylogram showing evolutionary relationships aligned with a character presence/absence heatmap. The tree branches are colored by the density of character changes (gene conversion events) inferred between nodes. The adjacent heatmap shows the character states for 172 events, with purple indicating presence, lilac indicating absence, and orange indicating ambiguity. Source data are provided as a Source Data file.

**Fig. 4:**
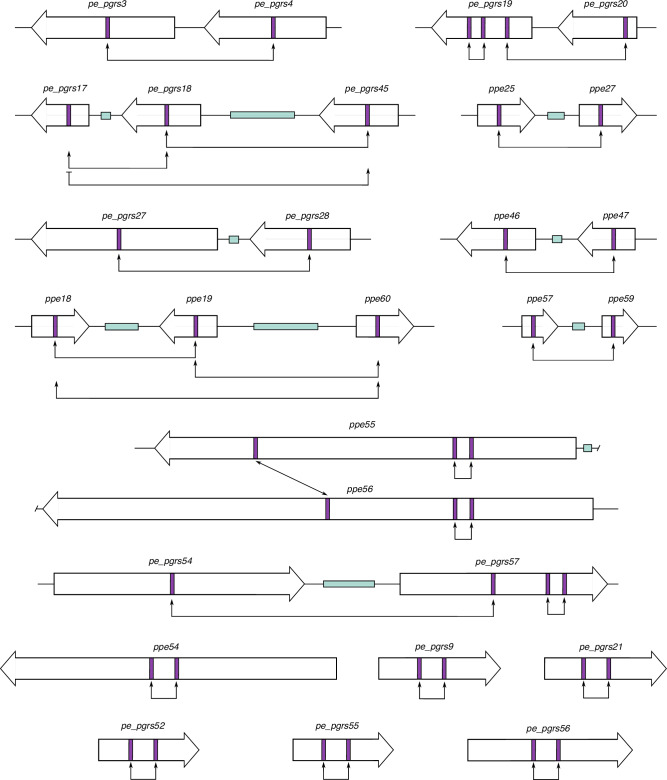
Schematic representations of gene conversion events between and within 28 *pe/ppe* genes. Each schematic represents a gene conversion event, illustrating the genes involved. Genes are depicted as thick arrows scaled to gene length and oriented according to their strand direction. Genes separated only by intergenic regions are connected by a thin line, while separation by additional loci is indicated by a blue bar. Intragenic events (within the same gene) are shown as two closely spaced red lines within the same arrow. Thin connecting arrows indicate the direction of gene conversion from donor to recipient genes. When arrows point in both directions, it indicates that those genes can function as both donors and recipients.

We generated MSAs for the 169 *pe/ppe* genes and measured their total nucleotide diversity. All genes were included in the analysis, with 109/169 (65%) genes present in all genomes (Supplementary Data 4). As shown in Fig. 3a, nucleotide diversity varied widely across the family, ranging from highly conserved to hypervariable genes. Furthermore, nucleotide diversity was unevenly distributed within individual genes (Supplementary Fig. 3). We accurately delineated 172 gene conversion events, which were detected in 28 *pe/ppe* genes, displaying diverse SNP patterns in terms of frequency and extent (Supplementary Data 5 and Fig. 3c). Validation with RDP5 confirmed all events except one in *pe_pgrs52*, which was confirmed by manual curation. A schematic overview of these events is provided in Fig. 4. Additionally, we identified gene conversion as the primary driver of nucleotide diversity (Fig. 3b).

The distribution of these gene conversion events was closely related to branch lengths (Fig. 3c) (Spearman’s rho = 0.84, *p* = 4.8 × 10^-5^, *n* = 16). Most events occurred only once, though some mapped to multiple branches of the tree (Fig. 3c). We quantified this convergent evolution by analyzing the number of parsimony steps on the short-read-based phylogeny (Supplementary Fig. 4a). A majority of these events align with the expansion of TB during the XVIII-XIX centuries (Supplementary Fig. 4b).

### A wide range of structural polymorphisms in locus *ppe38-71* detected by complete genomes

**Fig. 5:**
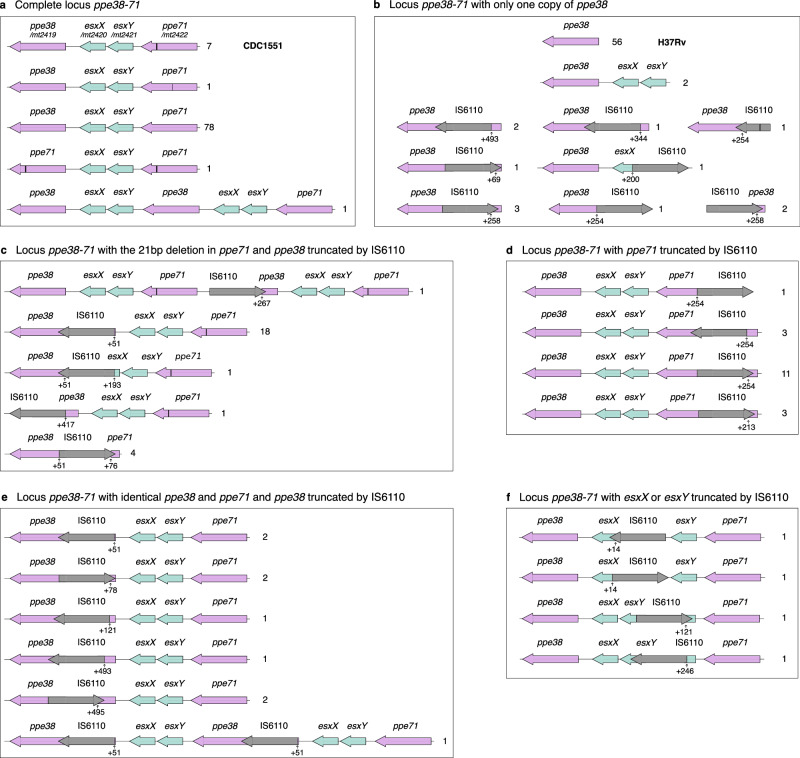
Schematic representation of polymorphisms of the *ppe38-71* locus. The *ppe38-71* locus includes *ppe38* (purple), *esxX* and *esxY* (blue), and *ppe71*, which may be identical to *ppe38* or contain a 21 bp deletion (indicated by a vertical black line). Due to a misannotation, the H37Rv reference genome (GenBank: AL123456.3) has only one copy of *ppe38*, but several H37Rv strains harbor the full functional locus. For this reason, we followed the annotation of the MTB CDC1551 reference genome (GenBank: NC_002755.2) to annotate this region in complete genomes, as recommended by Ates et al.^21^. Multiple polymorphisms of locus *ppe38-71* were identified in the dataset and grouped into six main categories: **a** complete locus; **b** single copy of *ppe38* with various truncations due to IS6110; **c** *ppe71* with a 21 bp deletion and *ppe38* truncated by IS6110; **d** *ppe71* truncated by IS6110; **e** *ppe71* identical to *ppe38*, with *ppe38* truncated by IS6110; **f** complete locus with *esxX* or *esxY* truncated by IS6110. The number of samples corresponding to each genotype is indicated on the right. Two samples lacked the entire locus. Most of these loci conformations have unknown consequences for virulence since they have not been previously reported. This is a salient example of how complete genomes can help to understand the evolution of traits such as virulence in MTB.

We were particularly interested in structural polymorphisms in the *ppe38-71* locus, as it is one of the most variable regions of the MTB genome, and it has been linked to the secretion of virulence factors such as PE_PGRS and PPE-MPTR proteins^21,22^. Inactivation of this locus by deletion or truncation of *ppe38* exhibits diverse effects on virulence in mouse models of infection^21,23^. We found substantial structural diversity in the *ppe38-71* locus even within our dataset enriched in lineage 4 (Fig. 5): *ppe38* was intact in 169/216 (78%) genomes, altered in 45/216 (20%), and completely absent in 2/216 (2%). Unexpectedly, three samples had duplications of the locus. Most of these locus conformations are newly described, and their impact on virulence remains unknown.

### T-cell epitopes of PE/PPE antigens are hyperconserved but can undergo gene conversion events

**Fig. 6:**
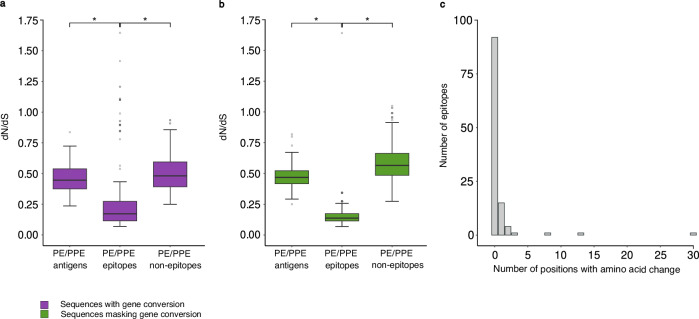
Evolutionary patterns of experimentally validated human T-cell epitopes from PE/PPE antigens in *M. tuberculosis.* **a** Comparison of dN/dS ratios across three different categories of PE/PPE antigens: 115 PE/PPE epitopes, the non-epitope regions of the same PE/PPE antigens, and 61 complete PE/PPE antigens. **b** Same comparison as the one shown in panel A, but analyzing the clonal frame of sequences exclusively (i.e., removing gene conversion events); Brackets indicate one-sided Wilcoxon Signed-Rank Tests of pairwise category comparisons; *p < 0.01. In (**a**), epitopes were significantly more conserved than both non-epitope regions (*p* = 4.1 × 10^−15^) and full antigens (*p* = 2.5 × 10^−16^). Similarly, within the clonal frame (**b**), epitopes exhibited significantly higher conservation compared to non-epitope regions and full antigens (both *p* < 2.2 × 10^-16^). In the box plots, the center line represents the median, and the box bounds represent the first and third quartiles (the interquartile range, IQR). The whiskers extend to the smallest and largest values (minima and maxima) that are within 1.5 times the IQR from the box bounds. Data points beyond this range are plotted individually as outliers. **c** Frequency distribution showing the PE/PPE epitopes grouped by the number of observed amino acid changes. Supplementary Data 6 contains epitope sequences and additional genomic data. Source data are provided as a Source Data file.

We investigated how intragenic variation affects *pe/ppe* antigen-encoding genes, focusing on T-cell epitopes, the part of antigens recognized by the immune system. We analyzed 61 experimentally confirmed human T-cell antigens encoded by *pe/ppe* genes (Supplementary Data 4). PE/PPE antigens exhibited similar nucleotide diversity (*p* = 0.15, two-sided Wilcoxon Rank-Sum Test) and were proportionally influenced by gene conversion events compared to other family members (Fig. 3b) (*p* = 0.67, Fisher’s Exact Test). PE/PPE epitopes were significantly more conserved than non-epitope regions (*p* = 4.1 × 10^-15^, one-sided Wilcoxon Signed-Rank Test) and full antigens (*p* = 2.5 × 10^-16^, one-sided Wilcoxon Signed-Rank Test), with a median dN/dS of 0.17 versus 0.48 and 0.45, respectively (Fig. 6a, Supplementary Data 6). However, certain epitopes from *ppe57, ppe19*, and *ppe18* underwent gene conversion events, accumulating many non-synonymous substitutions (Fig. 6c). After excluding gene conversion events, we recalculated the dN/dS ratio and observed the same pattern of epitope hyperconservation (Fig. 6b), despite comparable levels of variation in epitope and non-epitope regions (0.68% vs. 0.61% segregating sites, *p* = 0.38, Fisher’s Exact Test). These are strong indicators of purifying selection acting in epitope sequences. Regarding indels and structural variation in PE/PPE epitopes, we detected a small deletion (6 bp) affecting one epitope of *ppe18* in two isolates, and a large deletion (4670 bp) affecting one epitope of *pe_pgrs35* in one isolate.

### Complete genomes enhance genetic variant detection at a local scale and delineate recent transmission

**Fig. 7:**
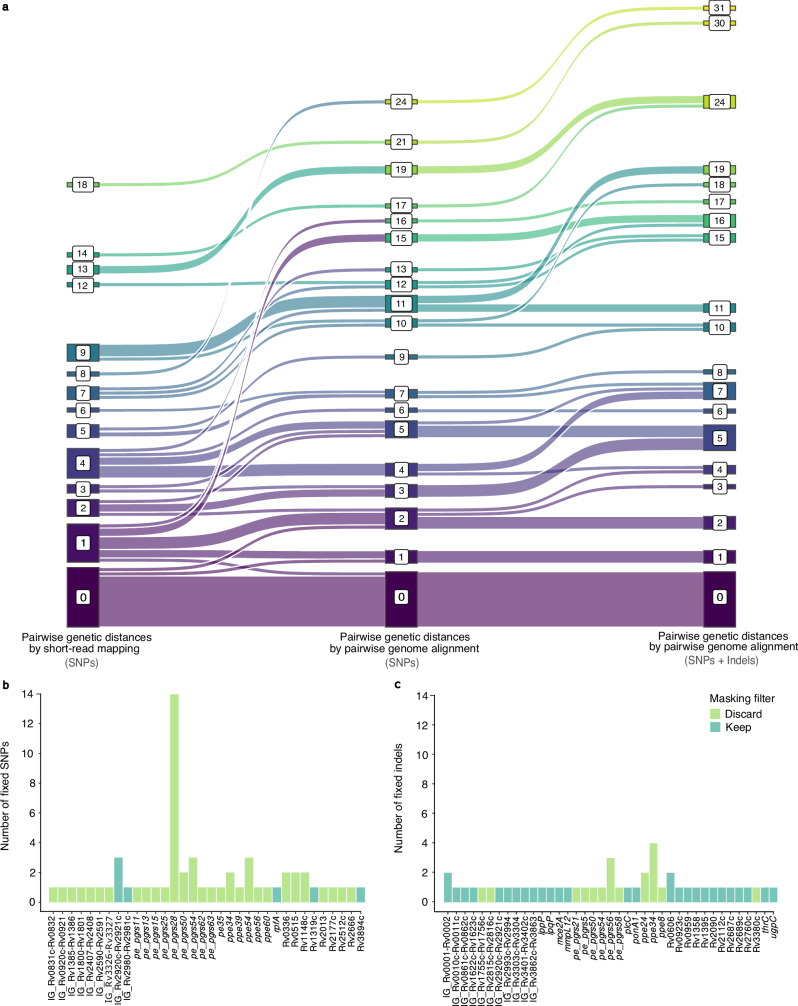
Gain of fixed mutations between closely related samples using complete genomes and their location in the genome. Pairwise genetic distances between samples at ≤20 SNPs according to short-read data were calculated by three approaches that included: i) short-read SNP data, ii) complete genome SNP data, and iii) complete genome SNP and indel data. **a** The Sankey diagram shows the increase in pairwise genetic distances when moving from short-read mapping to complete genome comparisons. **b, c** Frequency and genomic distribution of additional SNPs and indels, respectively, detected only by complete genome data. Source data are provided as a Source Data file.

We next focused on recently diverged isolates, analyzing pairs of isolates with a genetic distance of up to 20 SNPs, as determined by short-read data. Fifty-two pairs from 65 closely related isolates were examined in detail (Fig. 7a, Supplementary Data 7). On average, 2.5 SNPs per pair were gained, with a median of 1 (–1 to 16) SNP and 2 mutations (SNPs and indels). Upon manual inspection, we identified one SNP, detected by short-read mapping but not complete genomes, as a false positive. In 31/52 (60%) pairs, additional fixed SNPs were identified from the complete genome data, and fixed indels were found in 24/52 (46%) pairs. Overall, additional variation was detected in 36/52 (70%) pairs. Notably, 12 out of 14 pairs with 0 SNP distance remained unchanged, indicating that 0-SNP distances from short-read sequences are generally also reliable markers of very recent transmission. On the contrary, at 5 and 10 SNP thresholds, most pairs gain diversity considering only SNPs: 18/38 (47%) and 27/47 (57%) respectively; and when incorporating indels: 22/38 (58%) and 31/47 (66%) respectively. Furthermore, four pairs showed a substantially larger increase in SNPs (15 on average). A deeper investigation revealed that one isolate, common to all four pairs, underwent a gene conversion event between the *pe_pgrs27* and *pe_pgrs28* genes.

A total of 50/57 (88%) non-redundant SNPs detected only by complete genomes were in masked regions (Fig. 7b), with the *pe/ppe* gene family overrepresented as in the global analysis. The remaining 7/57 (12%) SNPs were found in unmasked regions but were missed by the short-read mapping analysis (Supplementary Note 3). The location of indels detected by complete genomes is shown in Fig. 7c. Next, we investigated whether masked variants could have been retained in the short-read mapping approach. A detailed list with 1081 polymorphic positions is provided in Supplementary Data 8. F1 score distributions highlighted the value of masking individual positions rather than entire regions (see Supplementary Note 3).

### Better genetic transmission network resolution is enabled by complete genomes over short-read mapping

We identified 24 genomic transmission clusters based on a 10-SNP threshold using the short-read mapping approach, with cluster sizes ranging from 2 to 5 cases (Supplementary Data 9). We then reconstructed the same clusters using complete genomes, analyzing SNPs, and SNPs combined with indels separately. Transmission network diagrams for all clusters and approaches are provided in Supplementary Fig. 5. Complete genome analysis revealed more information in 15/24 (62%) of the clusters, while 9/24 (38%) remained unchanged. Among the clusters with differences, 5/24 (21%) displayed a different topology, 2/24 (8%) showed changes in edge lengths, altering the relative distances but maintaining the underlying topology, and 8/24 (33%) only exhibited changes in edge lengths while maintaining the same relative distances and underlying topology. Regarding distances between samples in the network, 32/47 (68%) of links showed changes, with an average of 2.5 mutations gained per link and a median of 2 mutations (–1 to 11), corroborating the pairwise distance analysis above.

We detected indels in 6/24 (25%) of transmission clusters, with varying lengths and structural characteristics. The most common were medium-small indels, present in 8 isolates. Other structural variants included: 3 tandem duplications, 3 collapsed tandem repeats, 2 duplications, and 6 insertions of the IS6110 sequence. Additionally, we identified a gene conversion event from *pe_pgrs27* to *pe_pgrs28* that affected isolate G1823 in Cluster 10 (Supplementary Fig. 5). It involved 14 additional SNPs that were included as a single mutation event in the analysis.

### Cluster-specific reference genomes allow for the incorporation of non-fixed variants into transmission analyses

To evaluate the impact of using a closer reference genome instead of a common reference, we analyzed short-read sequencing data from 372 MTB isolates belonging to 77 clusters (ranging from 2 to 21 samples) collected in the Valencia Region between 2014 and 2019 (Supplementary Data 10). The comparison of both approaches demonstrated that using a closer reference genome provided greater reliability, particularly during the mapping step (see Supplementary Note 4 for details).

**Fig. 8:**
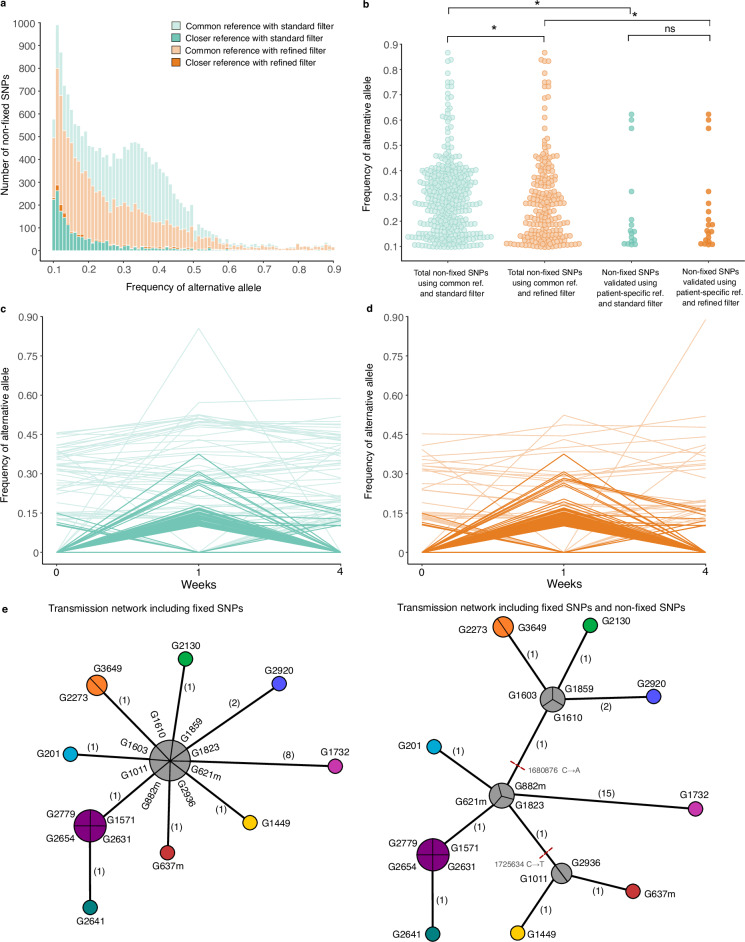
Differences between common and closer reference genome approaches. **a** Distributions of non-fixed SNPs detected in isolates from genomic transmission clusters. Our standard short-read pipeline was performed twice on 372 MTB samples of 77 transmission clusters, using both MTBCA and cluster-specific complete genomes as references. Standard and refined masking filters were applied to each reference approach. Distributions for the common reference with standard and refined filters are in pastel blue and pastel orange, respectively; cluster-specific reference results are in blue and orange. **b** Distributions of non-fixed SNPs in four patients with two serial isolates. The left panel shows total non-fixed SNPs detected by the common reference approach in pastel blue for the standard masking filter and in pastel orange for the refined one. The right panel displays the distributions of validated non-fixed SNPs by the patient-specific reference approach for both types of filters (Supplementary Table 2). Brackets indicate one-sided Wilcoxon Rank-Sum Tests of pairwise category comparisons; ns, not significant; *p < 0.05. Use of the standard filter with a common reference resulted in significantly higher AF distributions of non-fixed SNPs compared to the refined filter (*p* = 0.04). In contrast, no significant difference between filters was observed when using patient-specific references (*p* = 0.65). Comparisons between reference types also showed significant differences under both the standard filter (*p* = 0.01) and the refined filter (*p* = 0.05). Trajectories of non-fixed SNPs from isolates of the same patient along three time points. The figures show the non-fixed SNPs detected by the common reference approach using the standard masking filter in blue (**c**) and the refined masking filter in orange (**d**). The frequencies (y-axis) are shown at 0, 1, and 4 weeks (x-axis). Only the non-fixed SNPs validated by the patient-specific reference approach are colored in intense blue or orange. It shows that most mutations at intermediate frequencies could not be validated (Supplementary Table 2). **e** Example of an original transmission network (left) and the same network including non-fixed SNPs (right). This figure illustrates how incorporating non-fixed SNPs (indicated by red dashed lines) into transmission analysis can enhance network resolution. Source data are provided as a Source Data file.

Notably, and linked to the next section, mapping to a cluster-specific reference genome revealed a sharp reduction of non-fixed variation compared to our conventional pipeline relying on a common reference genome. We observed a ten-fold reduction in non-fixed SNPs (two-sided Wilcoxon Rank-Sum Test, *p* < 2.2 × 10^-16^), which resulted in a five-fold reduction when compared to using the refined masking filter with the common reference approach (two-sided Wilcoxon Rank-Sum Test, *p* < 2.2 × 10^-16^) (Fig. 8a). The ability to accurately detect genuine non-fixed variant calls using a closer reference genome enabled the incorporation of this source of variation into the reconstruction of 31/77 (40%) transmission clusters. We were able to enhance the epidemiological resolution of 18/77 (23%) clusters (example shown in Fig. 8e).

### Patient-specific reference genomes reveal genuine within-host variation in serial isolates

We next explored whether mapping short-read data to a patient-specific reference genome also enhances non-fixed variant calling in patients with serial isolates. We analyzed five cases for which a complete genome and matched short-read data were available, along with at least one additional isolate sequenced by short-read technology (Supplementary Data 10). Our results showed that 524/640 (82%) of non-fixed variants detected when mapping short reads to a common reference and applying the standard masking filter were false positives. A median of 5.1% (0 to 63%) non-fixed SNPs per patient were validated by the patient-specific reference approach (Fig. 8b, Supplementary Table 2). Notably, this was also observed in some non-fixed SNPs reaching a frequency of 0.85 (Fig. 8c, d). A refined filter partially ameliorates the issue of false positives. Still, 276/396 (69.7%) of non-fixed SNPs were false positives, and a median of 10.8% (0 to 78%) of non-fixed SNPs was validated (one-sided Wilcoxon Rank-Sum Test, *p* = 0.04) (Fig. 8b). This filtering effect was not observed when using the patient-specific reference genome (one-sided Wilcoxon Rank-Sum Test, *p* = 0.65); in fact, some non-fixed SNPs located in previously masked regions were recovered, suggesting that masking filters may not be necessary for this approach. These findings have major implications for interpreting previously published studies analyzing within-host variation.

## Discussion

In this study, we demonstrate that high-quality complete genomes provide a more comprehensive view of MTB diversity, with important implications for both pathogen evolution and epidemiology. By combining our optimized DNA extraction protocol with HiFi sequencing, we can generate highly accurate long-read data to construct complete genomes without relying on short-read polishing. Complete genomes significantly increase the observed genetic diversity at a global scale, uncovering previously overlooked variation that enhances our understanding of MTB evolution, particularly the role of *pe/ppe* genes in host-pathogen interactions. While the increase in diversity is less pronounced at the epidemiological scale, it still improves the resolution of transmission networks. On the contrary, we observe a substantial downward revision of within-host diversity relative to previous estimates.

MTB has been regarded as a monomorphic pathogen^24^. While this is largely still true, complete genomes depict considerably more variation than previous approaches, allowing the identification of diversity hotspots. Pairwise genome alignment comparisons reveal a median of 312 additional SNPs, with almost 90% of this variation occurring in the inaccessible regions. Consistently, complete genomes from L4 yielded a substantially 1.44-fold higher estimated evolutionary rate. Taken together, these observations suggest that the higher evolutionary rate inferred from complete genomes is partly driven by variation within complex, repetitive regions. Masking detectable gene conversion in *pe/ppe* genes reduces this difference to 1.25-fold, suggesting that this source of diversity strongly contributes to the accelerated evolutionary rate. However, a substantial fraction remains, consistent with additional contributions from undetected gene conversion in other repetitive gene families or from diversity hotspots detected in Fig. 2a. Our analysis suggests that complete genomes potentially refine molecular dating approaches and provide additional sources of variation to explore the role of MTB genetic diversity in functional or clinical phenotypes.

As expected, most of the observed diversity was concentrated in several *pe/ppe* genes, whose high variability has been linked to potential roles in antigenic variation and immune evasion. However, these hypotheses are unconfirmed due to conflicting evidence and difficulties in reconstructing the evolutionary history of the entire *pe/ppe* repertoire^6,16,25,26^. T-cell-mediated immunity is central for protective responses in TB^27,28^, with previous studies showing conservation of T-cell antigens in epitope regions^4,7^. Our results reveal that T-cell PE/PPE epitopes follow the same evolutionary pattern previously observed in non-PE/PPE antigens, with epitopes hyperconserved in comparison with non-epitopes and full antigen sequences. However, we observe gene conversion events introducing numerous amino acid changes in some epitopes of PPE18, PPE19, and PPE57 antigens, a key source of variation accurately captured by complete genomes.

Remarkably, we found extensive gene conversion in *ppe18*, a subunit of the M72/AS01E vaccine antigen, which showed 49% protection in a phase IIb trial^29^. While most events occur outside epitope regions, some affect epitope sequences as well. High genetic variability in *ppe18* has been described before^19,26,30^, but only complete genomes have revealed the full extent of this genetic diversity, making the *ppe18* region a diversity hotspot in MTB (see Fig. 2ab and Supplementary Fig. 3), and confirming that gene conversion, the leading mechanism of this variation, can also affect epitope regions. While T-cell-based responses are not the only contributor to protection^28^, our results highlight the need to evaluate the full diversity of antigens for vaccine development and the role of gene conversion in the evolution of these genes. Other structural variations may also have an impact on virulence, as illustrated by the extraordinary diversity found in the *ppe38-71* locus (Fig. 5).

At an epidemiological scale, our work establishes the first population-based benchmark for tracing genomic transmission by using complete genomes. Currently, genomic transmission links are defined by a 10–12 SNP threshold, and the low observed diversity precludes more detailed reconstruction of genomic transmission networks^31,32^. Complete genome data offer a modest but significant gain in genetic distance between closely related samples, with a median of 1 additional SNP and 2 additional mutations in pairwise comparisons. Even more, this approach allows the confident incorporation of indel data in transmission analyses. Although indels have been traditionally excluded due to detection challenges, there is growing interest in developing more robust methods for detecting and integrating them, as they represent a potentially valuable source of information^33,34^. In our study, we reliably identified indels and structural variations, recovering genetic signals that are typically captured by classical genetic markers such as IS6110-RFLP, spoligotyping, and MIRU-VNTR. Occasionally, multiple SNPs can be gained from gene conversion events (as shown in Cluster 10)^19^. The inclusion of additional diversity affects the underlying genetic networks and alters the reconstruction of 20% of transmission histories (see Supplementary Fig. 5).

While the increase in resolution is modest, it is important to note that the 0–5 SNP range yields the most actionable results for public health intervention. Consequently, a variation of even one or two SNPs can be decisive in determining whether an investigation is initiated. Furthermore, our analysis can provide stronger support for those cases with 0 SNP differences, improving the inferences derived from short-read approaches. Although the limited number of confirmed contact pairs in our current dataset precludes definitive conclusions, larger benchmarking datasets spanning a broader timeframe will help formally test this trend. While genetic network analysis is not definitive, improved resolution provides valuable insights for outbreak investigations, better guiding the allocation of public health resources.

Complete genomes also impact the understanding of within-host variation, which is typically detected by identifying non-fixed variants within a bacterial population^12^. Although short-read data can accurately estimate variant frequencies, distinguishing genuine variants is challenging due to errors in mapping and calling to a common reference genome^9,11,35^. Here, we show that mapping short reads to a complete genome from the same patient vastly improves non-fixed variant detection, removing up to 70–80% of variants from mapping to a common reference depending on the masking filter. This strong filter effect highlights the relevance of applying refined filters when using a common reference^5,36^. In contrast, minimal differences were observed when using a patient-specific reference, suggesting that masking filters may be unnecessary in this approach. Even in the case where closer references are not available, our dataset provides a robust repository of high-quality complete genomes from several lineages and sub-lineages of L4 that allows the selection of optimal references for future analyses.

These findings have profound implications for understanding the evolution of the pathogen during infection and treatment, and the emergence of drug resistance, calling into question previous estimates of within-host diversity. In addition, non-fixed variants may increase resolution in transmission networks^13,37,38^. In our population-based dataset, we could use them to increase the resolution in 23% of transmission clusters, suggesting that cluster-specific references can pave the way for integrating non-fixed variants to clarify outbreaks.

Currently, long-read sequencing is not without caveats as it requires higher DNA quality and quantity than short-read sequencing, which is difficult to meet with mycobacteria. However, these requirements are expected to lessen shortly; meanwhile, we provide a fine-tuned protocol that has allowed us to recover complete genomes in almost all cases. Streamlining downstream analysis of complete genomes is also critically important; accurate multiple genome alignment remains a challenge^39^, especially for structural variants or high-complexity regions such as certain *pe*/*ppe* genes. We also acknowledge that the high enrichment of Lineage 4 in our dataset restricts the representation of global diversity. Even so, it still offers a more detailed view of MTB genetic diversity across evolutionary scales and reveals the full potential of complete genomes. Future work with more diverse samples is needed to elucidate the role of different sources of diversity in the MTBC.

In summary, we present the first large-scale genomic study of *M. tuberculosis* using complete genomes across evolutionary scales. Advances in long-read sequencing technologies suggest that de novo genome assembly will likely become routine in the future, and the resulting opportunities will present challenges in analysis and interpretation. Our work reveals substantial untapped genetic diversity that provides new avenues for understanding the ongoing evolution of *M. tuberculosis* and how it is shaped by host-pathogen interactions. Furthermore, improving resolution at both the epidemiological and within-host levels will support the development of new strategies for TB control.

## Methods

### Study design

We performed long-read sequencing on all MTBC culture-positive samples collected as part of a local population-based genomic study in the Valencia Region (Spain) from January 2016 to December 2016. Short-read sequencing data of MTBC isolates collected from January 2014 to December 2016 were previously generated and published on the European Nucleotide Archive (ENA) in the BioProjects PRJEB29604, PRJEB38719, PRJEB65844, PRJEB89397, and PRJEB70424. Isolates from January 2017 to December 2019 were newly sequenced following the methodology described in Cancino-Muñoz et al.^40^, and can be found under BioProject accession PRJEB89456. Only one representative sample from each patient was included in all the analyses except for the within-host diversity analysis, in which we analyzed serial short-read sequences from five patients (see below). Serial isolates for this analysis were deposited on BioProjects accessions PRJEB89397 and PRJEB65844.

Description of the underlying study where samples were obtained can be found in Cancino-Muñoz et al.^40^. Approval for the study was given by the Ethics Committee for Clinical Research from the Valencia Regional Public Health Agency (Comité Ético de Investigación Clínica de la Dirección General de Salud Pública y Centro Superior de Investigación en Salud Pública, Reference 20210430/03).

### High-quality, high-integrity genomic DNA extraction and long-read sequencing

To determine if available genomic DNA samples from 2016 were suitable for long-read sequencing, we analyzed the requirements established by the manufacturer. We measured the DNA concentration using the Qubit® 3.0 Fluorometer (Thermo Fisher Scientific Inc., Waltham, Massachusetts), the DNA Integrity Number (DIN) using the TapeStation system (Agilent Technologies Inc., Santa Clara, CA), the A260/A280 and A260/A230 ratios using Tecan’s NanoQuant Plate® (Tecan Trading AG, Switzerland), and the presence of RNA by comparing the concentration of DNA detected by Qubit and NanoQuant. Isolates for which the genomic DNA did not meet the requirements were grown in solid media from the frozen culture for three weeks at 37 °C. We optimized the standard CTAB-based DNA extraction protocol to obtain high-quality, high-molecular-weight genomic DNA. Finally, all the DNA samples received RNase treatment (60 min at 37 °C) to remove contaminant RNA and were purified using magnetic beads (0.45x ratio) to select the longest DNA fragments. The step-by-step optimized protocol can be found in protocols.io^41^. Long-read sequencing libraries were generated using the SMRTbell® Express Template Prep Kit 2.0 (Pacific Biosciences of California Inc., Menlo Park, CA), following the manufacturer’s protocol. PacBio HiFi sequencing was performed using the Sequel II (SQII) platform. HiFi reads were obtained using the Circular Consensus Sequencing (CCS) mode on the SMRT Link Software (Pacific Biosciences of California Inc., Menlo Park, CA).

### Assessment of long-read HiFi sequencing

Quality control of HiFi reads was performed using a customized bash script to determine basic statistics and LongQC (v1.2.0b)^42^. Before de novo assembly, long reads were classified by taxonomy with Kraken (v0.10.5)^43^ using an ad-hoc database^44^, and those not belonging to the MTBC were removed with KrakenTools (v1.2)^45^ for decontamination. HiFi reads containing the remaining PacBio adapter sequences were removed with HifiAdapter (v2.0.0)^46^.

Additionally, we determined the single-mismatch rate per base in HiFi reads. Long reads from each isolate were aligned to the inferred MTBC most likely common ancestor (MTBCA)^8^ reference genome with minimap2 (v2.26)^47^. Unmapped reads and secondary and supplementary alignments were discarded. The number of single mismatches per read was obtained using the pysam^48^ module through a customized Python script^49^. Finally, the error rate per base was calculated by normalizing the number of mismatches to the number of aligned bases per read and then averaging across all reads.

### De novo assembly and manual curation

Assemblies from HiFi reads were built using Flye (v2.9.2)^50^ with two polishing iterations and circularized using Circlator (v1.5.5)^51^. To identify misassemblies caused by collapsed or expanded repeats, long reads were mapped to the assemblies with pbmm2 (v1.13)^52^, and genome coverage was plotted using the package ggplot2^53^ from R (v4.2.2)^54^. Regions whose coverage was significantly higher or lower than average were visually inspected using Artemis (v18.2.0)^55^, and assembly errors were manually corrected. Finally, since single-base substitutions and short indel errors can occur during the generation of draft genome assemblies, final consensus sequences were polished using their long reads. The polishing process involved three steps: i) mapping long reads to the assembly with pbmm2 (v1.13)^52^; ii) calling variants with Freebayes (v1.3.6)^56^ and normalizing using vt (v0.57721)^57^; and iii) correcting positions in the assembly with the consensus allele (frequency above 0.5) using a customized Python script^49^. Finally, genome annotation was lifted over from the H37Rv reference genome (GenBank accession: AL123456.3) to each assembly, employing Liftoff (v1.6.3)^58^ with -copies and -overlap 0.2 parameters and blastn (v2.12.0 + )^59^ for complicated genomic regions.

### Quality control of genome assemblies

We evaluated three dimensions for a comprehensive assembly quality assessment: contiguity, completeness, and correctness^60^. The methods used in this analysis are reference-free because distinguishing between assembly errors and genuine biological differences is challenging and can lead to misinterpretation of results^61^.

Contiguity reflects how well the assembled sequence covers the underlying genome and was assessed using the following metrics: number of contigs, total length, circularization, and N50.

Completeness evaluates the gene content of the assembly and was measured with: i) BUSCO (v5.5.0)^62^ applying the Corynebacteriales database to identify the presence or absence of highly conserved genes, ii) IDEEL^63^ to determine the proportion of predicted proteins that are at least 95% the length of their best-matching known protein in a database, and iii) long-read mapping coverage and the proportion of mapped/unmapped reads, iv) Merqury (v1.3)^64^ to calculate k-mer completeness using short reads, v) Prodigal (v2.6.3)^65^, to measure the average length of predicted proteins, and vi) QUAST (v.5.2.0)^66^ to obtain the GC content.

Correctness concerns the accuracy of each base pair in the assembly and how accurately contigs represent the genome sequence. To assess this feature, we measured: i) the concordance between the assembly and short-read data by mapping short reads against the assembly to calculate horizontal coverage and proportion of mapped/unmapped reads and detect small mismatches with Freebayes (v1.3.6)^56^, ii) the presence of high frequency (above 0.9) misassemblies detected by Sniffles2 (v2.3.3)^67^ using long reads, and iii) the assembly quality value (QV) by Merqury (v1.3)^64^, which measures the base-level accuracy using short-read k-mers.

### Multiple Genome Alignment from Complete Genomes

We obtained a Multiple Genome Alignment (MGA) that included 216 complete genomes and the MTBCA reference genome using Minigraph-Cactus (v2.8.4)^68^ with default parameters. The final MGA, originally in HAL (Hierarchical Alignment) format, was converted to MAF (Multiple Alignment Format) format using cactus-hal2maf with the parameters dupeMode single and noAncestors from HAL tools (v2.3)^69^. Synteny blocks in the MGA were identified using maf2synteny (v1.2)^70^.

The complexity of MGA algorithms is known to affect alignment accuracy. To minimize the risk of false positive variant calls, we refined the initial MGA by masking ambiguous positions. These were defined as positions with single variants not supported by at least one of two pairwise genome alignment approaches (nucmer (v4.0.0rc1)^71^ or minimap2 (v2.26)^47^). First, we obtained pairwise variant calls between the MTBCA reference genome and each sample from the MGA in MAF format using a customized Python script^49^. In parallel, pairwise alignment and variant calling were performed between the MTBCA reference genome and each complete genome with two different tools: i) nucmer (v4.0.0rc1)^71^ with the maxmatch parameter and dnadiff (v1.3)^72^ for variant calling. ii) minimap2 (v2.26)^47^ with the asm20 parameter and paftools (v2.26)^47^ for variant calling. A position was masked in a particular sample of the MGA when it harboured a variant only detected in the global alignment. If the number of samples with a variant in a specific position differed by more than 10% between the three approaches, the position was masked in all the samples of the MGA.

Additionally, positions with two or more alleles at frequencies between 0.1 and 0.89 in each complete genome were individually masked in the MGA, ensuring that only fixed variants were included in the downstream analyses, defined here as mutations with an allele frequency >0.9 in the culture of each MTB isolate.

### Reference-mapping genomic analysis using short reads and phylogenetic analysis

We conducted a standard bioinformatic analysis to analyze short-read data, applying a validated pipeline^73^ that performs comparably to those developed by major Public Health TB reference laboratories. The workflow overview is: i) Read trimming and filtering, ii) Read decontamination with Kraken (v0.10.5)^43^, iii) Mapping MTBC short reads to the MTBCA reference genome using BWA mem (v0.7.10)^74^, iv) Variant calling with VarScan2 (v2.3.7)^75^ and GATK HaplotypeCaller (v3.8)^76^. Single nucleotide polymorphisms (SNPs) called with high confidence are categorized into: fixed SNPs with allele frequency (AF) equal to or above 0.9, and non-fixed SNPs with AF between 0.1 and 0.89, and iii) Annotation of variants using SnpEff (v4.1)^76,77^ with H37Rv as reference (GenBank accession: AL123456.3) and masking of repetitive regions such as PE/PPE family and mobile elements. Lineage typing was performed by comparing SNPs with a catalog of phylogenetic marker positions^73^. Drug resistance profiles were determined by identifying mutations associated with resistance, as listed in the first version of the World Health Organization (WHO) catalogue^78^, and considering the expert rules. In line with a previous study assessing genomic resistance prediction in our setting^79^, isolates carrying the *inhA* S94A mutation were classified as resistant. Specific parameters for each step can be found at the following repository: http://tgu.ibv.csic.es/?page_id=1794.

The reconstruction of transmission networks using short reads was performed in two steps: i) Construction of multiple sequence alignment (MSA) files with concatenated fixed SNPs and discarding invariant positions with snp-sites (v2.5.1)^80^, and ii) Construction of the genomic network with the MSA files applying a median-joining network inference method implemented in PopArt Software (v1.7)^81^.

### Comparison between complete genomes and short-read data

Pairwise genetic distances between all pairs of samples were calculated from the refined MGA by applying a customized Python script^49^. As for short-read data, pairwise genetic distances were calculated with the R ape^82^ package from an MSA file with concatenated SNPs in unmasked genomic regions. In both cases, only fixed SNPs were considered. The correlation between pairwise genetic distances from both short-read and complete genome data (*n* = 23220) was determined using the Spearman rank coefficient.

Additionally, we compared the phylogenetic topology between the short-read-derived and the complete-genome-derived phylogeny. Phylogenies were inferred using the maximum likelihood method with the GTR evolutionary model in IQ-TREE2 (v2.2.5)^83^, applied to both the MSA from the short-read data and the refined MGA from the complete genome data. We then compared these two phylogenies using the Shimodaira-Hasegawa (SH) test in IQ-TREE.

After confirming that both trees were topologically congruent, we assessed the accumulation of mutations in each branch of the phylogeny (branch lengths), comparing the short-read MSA alignment to the long-read MGA alignment. We then used the tree derived from short reads as a reference to map the polymorphic positions from the complete genome MGA using TreeTime (v0.11.4)^84^. We correlated the branch lengths derived from the short-read alignment to the branch lengths from the complete genome alignment (*n* = 430), applying the Spearman Rank Correlation Test.

### Estimation of evolutionary rates

Rather than estimating the evolutionary rate of *M. tuberculosis* per se, this analysis focused on comparing evolutionary rate estimates derived from short-read mapping data, excluding complex, repetitive regions, and from complete genomes, under a consistent modelling framework.

Bayesian phylogenetic analyses were conducted using BEAST (v2.7.7)^85^ to estimate evolutionary rates from *M. tuberculosis* lineage 4 samples (*n* = 209), using short-read sequences and complete genomes independently. The site model was set to GTR + Γ4 with empirical base frequencies in BEAUti. A strict molecular clock was applied with a LogNormal prior on the evolutionary rate (M = 8.0, S = 1.2, in real space), and an initial value of 4.6 × 10⁻⁸ substitutions/site/year, following Bos et al.^86^. A coalescent constant population model was selected for the tree prior, with a 1/x prior distribution on population size.

As the dataset was homochronous, an ancient DNA sequence from a 17th-century Bishop (1679 AD)^87^ was included to calibrate the molecular clock in the short-read sequences analysis. For the complete genome analysis, the posterior time to the most recent common ancestor (tMRCA) from the short reads analysis was used as a prior, modeled as a normal distribution under three scenarios: (i) a weakly informative diffuse prior (mean = 200, SD = 150) covering a broad range beyond the short-read 95% HPD; (ii) an intermediate prior (mean = 300, SD = 100) centered on the short-read data tendency with expanded variance; and (iii) an informative prior (mean = 316, SD = 20) constrained to the short-read posterior median. The three Normal prior scenarios were chosen to span increasing levels of uncertainty around the short-read-derived tMRCA posterior, and to test robustness to prior specification. Ascertainment bias was corrected by defining invariant positions in the XML file, as recommended^88^. The short-read analysis was parameterized using an adjusted genome length, accounting for the exclusion of difficult-to-map positions.

For each dataset, three independent Markov chain Monte Carlo (MCMC) runs were conducted with chains of length 50 million and 10 million for each short-read and complete genome scenario, respectively. Convergence and mixing were assessed using Tracer (v1.6)^89^, with effective sample size (ESS) values > 200 considered acceptable following a 10–20% burn-in. Posterior distributions of the evolutionary rate from each run were visualized using the R packages ggplot2^53^, tidyverse^90^, and ggridges^91^.

Finally, to assess the contribution of gene conversion to the higher evolutionary rate estimated from complete genomes, we repeated the complete genome analysis after masking in the MGA the positions in *pe/ppe* genes with evidence of gene conversion (see below for details on gene conversion identification).

### Genetic diversity across the complete MTBC genome

To describe genomic diversity in our dataset at global and local scales, we measured two parameters: i) the number of segregating sites (S) to assess global genetic diversity accumulated since the emergence of MTB by comparing to the MTBCA reference genome, and ii) nucleotide diversity (π) to describe intrapopulation genetic diversity accumulated between extant strains of the MTB. A sliding window analysis was performed across the refined MGA, with segments of 200 bp at 1 bp intervals, using Variscan (v2.0.5)^92^. Regions with sequence coverage below 85% were excluded, and only windows with a size greater than one standard deviation below the mean were included. The number of segregating sites was normalized by window size to consider large gaps in the MGA. Regions with significantly higher diversity were identified by calculating the z-score for normalized S and π in each window. Then, genes and intergenic regions in windows with a z-score greater than five were annotated. The distribution of both the number of segregating sites and nucleotide diversity was plotted using the ggplot2^53^ package included in R (v4.2.2)^54^.

In addition, we performed a gene-enrichment analysis to identify Clusters of Orthologous Groups (COGs) that were overrepresented in our list of genes with higher diversity. Each gene was assigned to a specific MTB COG category^93^. We performed a Fisher’s Exact Test for each COG, and adjusted *p*-values were obtained by applying the Benjamini-Hochberg correction.

### In-depth analysis of the *pe/ppe* gene family

As *pe/ppe* genes are highly complex regions of the MTB genome, we analyzed them separately. The single *M. bovis* strain was excluded from this analysis. We extracted sequences of *pe/ppe* genes from each complete genome based on the gene coordinates in the H37Rv annotation (Genbank: AL123456.3). We then constructed an MSA of each gene with MACSE (v2.07)^94,95^, which aligns protein-coding sequences accounting for frameshifts, using the parameters -gc_def 11, -fs_term 20.0, and -fs 40.0. Sequences with at least 80% coverage and identity were included to reliably identify homologous positions for generating alignments. MSAs were curated with Aliview (v.2021)^96^ and manually corrected when needed.

Nucleotide diversity was measured for each gene using Variscan (v2.0.5)^92^. The analysis included all genes, even those not identified in all samples. Only positions in at least 85% of sequences in the MSA were considered.

To study structural variation in the locus *ppe38-71*, we identified *ppe38*, *esxX*, *esxY*, and *ppe71* using blastn (v2.12.0 + )^59^ from their annotation in the CDC1551 reference genome (Genbank: NC_002755.2, mt2419-22) as suggested by Ates, 2020^21^, and the GenBank X17348.1 sequence for the mobile element IS6110.

### Identification of gene conversion events in the *pe/ppe* gene family

To describe gene conversion events within the *pe/ppe* family, we first detected SNP clusters in each *pe/ppe* gene MSA relative to the MTBCA reference genome, manually curated. The limited genetic diversity of MTB allows clear identification of proximal variants. We then followed the approach suggested by Stritt et al.^19^: we used blastn (v2.12.0 + )^59^ with default parameters to align the targeted region against its complete genome and the MTBCA reference (a genome without evidence of gene conversion in the region analyzed). We identified a gene conversion event when obtaining two complete 100% identity hits against the complete genome, but only one against the MTBCA reference genome, which pinpointed the ‘donor’ sequence location. Both the targeted and the source regions are annotated by lift over as described in the previous section, confirming that both are aligned against the reference genome, thus discarding the possibility of duplications. In the case of gene conversion within a gene, tandem and segmental duplications were discarded by the visualization of the MSA with Aliview (v.2021)^96^.

Gene conversion was further confirmed using the RDP5 software^97^. We generated a new MSA for each gene, including only haplotype sequences. As the low genetic diversity of some *pe/ppe* genes can restrict the recombination signal needed by tools included in RDP5, and considering that in many cases the donor sequence is not in the same alignment, we aligned haplotype sequences from donor and receptor genes with MACSE (v2.07)^94,95^ when needed. We used the full set of tools included in RDP5 to detect recombination, with default parameters, particularly considering the GENECONV method^98^.

We traced the occurrence of the gene conversion events across the phylogeny (derived from short read data as previously described) using Mesquite (v.4.02)^99^, applying the Maximum parsimony method. We generated a combined phylogram and heatmap using the Python packages Biopython^100^ and Matplotlib^101^, coloring tree branches to reflect the density of event state changes occurring between nodes. To analyze the distribution of events relative to branch lengths, branch lengths were binned into 20-SNP intervals. For each interval (*n* = 16), we calculated the aggregate sum of both the branch lengths and the observed events to generate a frequency distribution. The relationship between these two variables was assessed using a Spearman’s rank correlation test.

Finally, to date the gene conversion events, we leveraged the dated phylogeny from L4 obtained when estimating the evolutionary rate with BEAST (see above Methods) and performed the same reconstruction analysis.

### Evolutionary analysis of T-cell epitopes of the PE/PPE antigens

We accessed the Immune Epitope Database (IEDB)^102^ (https://www.iedb.org/) to obtain all the T-cell epitope-encoding sequences of the *pe/ppe* gene family on January 16, 2025. We applied the following search parameters: linear peptide, organism *Mycobacterium tuberculosis* (ID:1773), T-cell assays, any MHC Restriction, human host, and infectious disease. We retrieved 264 epitopes of the *pe/ppe* family. We performed a tblastn (v2.12.0 + )^59^ against the H37Rv reference genome to identify the epitope’s genomic coordinates. After removing those with no hits found (40), identity below 80% (4), hits in a different gene from the one indicated in the database (4), and duplicates (6), 210 epitope sequences remained. Of those, 124 overlapping epitope sequences were merged into a single large sequence to generate non-overlapping epitope sequences. Finally, we generated 115 non-overlapping epitope sequences belonging to 61 *pe*/*ppe* genes.

We adopted the methodology described by Comas et al.^4^ to assess evolutionary signatures in *pe/ppe* genes. Briefly, sequences were classified into three categories: epitope regions, non-epitope regions, and complete antigen sequences. For each category, MSAs were generated by extracting the corresponding regions from the *pe/ppe* MSAs described above and concatenating them using FastaCon^103^. The number of synonymous and non-synonymous substitutions was then estimated using SNAP (v2.1.1)^104^, which implements the Nei and Gojobori method^105^. For each concatenated alignment, individual sequences were compared pairwise against the MTBCA reference genome to obtain dN/dS values. Median dN/dS ratios were subsequently calculated across all pairwise comparisons for each category and compared using the two-sided Wilcoxon Signed-Rank Test. In addition, the same analyses were repeated for epitope, non-epitope, and complete antigen sequences after masking gene conversion events^106^.

Finally, to detect indels and structural variants in PE/PPE epitopes, we identified gaps in the epitope MSAs, either in the MTBCA reference (indicative of insertion-like variants) or in the complete genomes (indicative of deletion-like variants). Putative variants were then classified using Nucdiff (v2.0.3)^107^ with default parameters by aligning the MTBCA reference to the corresponding complete genome.

### Pairwise analysis of genomically closed samples using complete genomes

We used short-read data to identify MTB pairs at genetic distances of up to 20 fixed SNPs, thus capturing recent and older transmission events according to conventional short-read pipelines. For each pair of closely related samples, pairwise alignments and variant calling using complete genomes were performed with the varifier (v0.4.0)^108^ make_truth_vcf function, which uses dnadiff (v1.3)^72^ and minimap2 (v2.26)^47^ to build a consensus call. To detect potential false negative SNP calls, alignments were manually inspected. Positions with a non-fixed SNP were masked in each assembly. Only fixed indels (with AF above 0.9) in each assembly were included. Indels detected in homopolymer sequences (three or more nucleotides) were excluded, as long-read sequencing technologies are more error-prone in these regions, making it challenging to discern between sequencing errors and real variation.

### Multiple genome alignments to reconstruct transmission networks with complete genomes

Individual MGAs were constructed using progressiveMauve (v2.4.0)^109^ with default parameters using complete genomes, for each transmission cluster previously identified by short-read data, applying a 10-SNP threshold. Mutations were extracted from MGAs using the msa2vcf tool from jvarkit^110^ and validated with pairwise alignment data. Only the variants that met the criteria specified in the previous section were included in the network analysis. SNP and indel data were used to reconstruct transmission networks by applying the median-joining network inference method with PopArt Software (v1.7)^81^. Additionally, in one cluster, we identified a gene conversion event between two genes belonging to the *pe/ppe* family. This recombination event was considered one mutation event in the network’s construction.

Once we identified the indels between samples of the same cluster, we aimed to clarify whether they were medium-small indels or structural variations. We performed pairwise genome alignments between all pairs of samples in transmission using Nucdiff (v2.0.3)^107^ with default parameters. Additionally, to identify whether some duplications were insertions of the mobile element IS6110 (Genbank accession: X17348.1), we compared them using blastn (v2.12.0 + )^59^.

### Assessment of variant calling from short-read sequencing data in masked regions

To evaluate the performance of variant calling from short-read mapping in regions systematically excluded from standard analyses, we assessed each masked position with an alternative genotype, using complete genomes as the ground truth. We detected ground truth SNPs by performing variant calling on the refined MGA, comparing the MTBCA reference genome with each complete genome using a customized Python script to parse the MAF file and identify variants relative to the ref. ^49^. Positions that were not validated during the refinement of the MGA for each sample were excluded from these measurements.

Variant calls from short-read data were obtained using the pipeline described earlier. In both complete genome and short-read mapping analyses, we focused exclusively on fixed SNPs located in regions discarded by the annotation filter. Agreement between the two methods was assessed by calculating recall (the proportion of ground truth variants correctly identified by short-read mapping), precision (the proportion of true variants among the variants detected by short-read mapping), and F1 score (harmonic mean of recall and precision) for each polymorphic position detected in at least 10 samples.

### Cluster-specific reference genomes for enhancing epidemiology analyses

The standard bioinformatic and downstream phylogenetic analyses were performed using the pipeline described above on short-read data from all MTBC isolates collected in the Valencia Region from 2014 to 2019. We selected the transmission clusters (applying a 10-SNP threshold) with at least one isolate from our dataset of complete genomes, allowing us to designate one of them as a cluster-specific reference genome. If more than one complete genome was available, we chose the one from the patient who was diagnosed first. We then reanalyzed each transmission group twice, using a different reference genome for each analysis: one with the cluster-specific complete genome and the other with the MTBCA as the reference genomes. We then compared basic indicators of short-read mapping quality. Additionally, both analyses were performed using two filters: i) a standard, annotation-based filter from Comas et al.^4^, and ii) a refined version adapted from Marin et al.^5^ that keeps regions with good mappability and base recall. Both filters are available in our repository: https://gitlab.com/tbgenomicsunit/Publications_resources.

Non-fixed SNPs, defined as calls with AF between 0.1 and 0.89 (inclusive), were analyzed in detail by comparing their distribution between the different reference and filter approaches. The cluster-specific reference genome approach allowed us to incorporate non-fixed variants that were fixed in another sample within the cluster into transmission analyses. To ensure the reliability of these non-fixed calls, we applied stricter criteria, excluding all supplementary alignments and reads without a proper pair mapped^12,111^ from the short-read alignment before the variant calling. As an indicator of improved epidemiological resolution, we assessed: i) clusters with changes in topology, and ii) clusters with changes in relative distances between samples but maintaining the topology.

### Patient-specific reference genomes to study within-host evolution

To study the impact of using a patient-specific reference genome for detecting within-host diversity, we analyzed five clinical cases from the Valencia Region in 2016. For each patient, we had a complete genome of the first isolate along with matched short-read data, as well as at least one additional isolate with short-read data obtained at least one week later. In one case, a third isolate was also available. We performed the previously described bioinformatic analysis using the MTBCA and a patient-specific complete genome as references. In addition, we conducted the analysis twice for each reference approach, using both the standard and the refined masking filters. We then identified non-fixed SNPs across all isolates and assessed the proportion of non-fixed calls detected by the common reference approach that was validated by the patient-specific reference approach.

### Reporting summary

Further information on research design is available in the Nature Portfolio Reporting Summary linked to this article.

## Supplementary information

Supplementary Information

Description of Additional Supplementary Files

Supplementary Data 1

Supplementary Data 2

Supplementary Data 3

Supplementary Data 4

Supplementary Data 5

Supplementary Data 6

Supplementary Data 7

Supplementary Data 8

Supplementary Data 9

Supplementary Data 10

Reporting Summary

Transparent Peer Review file

## Source data

Source Data

## Data Availability

All data generated or analysed during this study have been deposited in the European Nucleotide Archive under accession codes PRJEB29604, PRJEB38719, PRJEB65844, PRJEB89456, PRJEB89397, PRJEB70424, and PRJEB89421 and are publicly available. The accession numbers for each sample are included in Supplementary Data 1, 2, and 10. Source data are provided with this paper.

## Code availability

All original code has been deposited at Zenodo with 10.5281/zenodo.15489014 and is publicly available at https://github.com/anamatgu/MTBC_complete_genomes^48^.
