# Supplementary material for: Complete genomes reveal a refined map of Mycobacterium tuberculosis genetic diversity across evolutionary scales
Source: Nat Commun. 2026 Jun 6;17:7242. doi: 10.1038/s41467-026-73869-5 (PMC13396471; doi:10.1038/s41467-026-73869-5)
Supplement: Supplementary file 2 — Description of Additional Supplementary Files [file 41467_2026_73869_MOESM2_ESM.pdf]

Description of Additional Supplementary Files:

Supplementary Data 1: Basic statistics of long and short-read sequencing of 216 MTBC isolates. Sample IDs, bioproject, accession numbers, total number of reads, mean and median depths, and horizontal coverage are reported for long- and shortread sequences. Additionally, mean read length, N50 of reads, and % of GC content are reported for long reads.

Supplementary Data 2: Quality control of 216 MTBC complete genomes. Sample IDs, bioproject, accession numbers, and contiguity, completeness, and correctness parameters are reported for genome assemblies.

Supplementary Data 3: Genetic diversity hotspots across the MTB genome. Diversity measure, locus, gene, feature, start and end coordinates, strand, annotation filter, essentiality, and COG are reported for each genetic diversity hotspot identified.

Supplementary Data 4: Features of the pe/ppe gene family. Name of the gene, locus, start and end coordinates, number of segregating sites, nucleotide diversity, number of samples with the gene, and condition of encoding antigen and evidence of gene conversion are reported for each *pe/ppe* gene.

Supplementary Data 5: Matrix of gene conversion events in *pe/ppe* genes. Each event is described by indicating the receptor gene where it was identified, the number of the event in the gene (EX), and the location of the event in the alignment based on MTBCA coordinates. 0 indicates absence, 1 indicates presence, and - indicates absence of the gene in the complete genome.

Supplementary Data 6: Features of PE/PPE epitopes from the Immune Epitope Database. ID, protein sequence, length, genomic and gene start/end coordinates, name of the gene and locus, and number of amino acid changes are reported for each PE/PPE epitope.

Supplementary Data 7: Pairwise genetic distances between closely related samples. Samples ID, SNP distance from short-read data and complete genome data, indels from complete genome data, and the total number of mutations are reported for each pair of samples

Supplementary Data 8: Assessment of variant calling in positions masked by the shortread mapping approach. Genomic position, number of samples, FP, FN, TP, number of samples with ambiguous position, recall, precision, F1 score, gene, and genomic coordinates are reported.

Supplementary Data 9: Genetic distances between isolates of the same transmission network. Cluster ID, Samples ID, SNP distance from short-read data and complete genome data, indels from complete genome data, and the total number of mutations are reported for each pair of samples

Supplementary Data 10: Cluster and patient-specific reference genomes and related samples. Cluster and patient IDs, sample ID, reference genome, project accession, and sample accession are reported.
