# Supplementary material for: Complete genomes reveal a refined map of Mycobacterium tuberculosis genetic diversity across evolutionary scales
Source: Nat Commun. 2026 Jun 6;17:7242. doi: 10.1038/s41467-026-73869-5 (PMC13396471; doi:10.1038/s41467-026-73869-5)
Supplement: Supplementary file 13 — Reporting Summary [file 41467_2026_73869_MOESM13_ESM.pdf]

nature portfolio

Corresponding author(s): Iñaki Comas

Last updated by author(s): Apr 27, 2026

Reporting Summary

Nature Portfolio wishes to improve the reproducibility of the work that we publish. This form provides structure for consistency and transparency in reporting. For further information on Nature Portfolio policies, see our Editorial Policies and the Editorial Policy Checklist.

Statistics

For all statistical analyses, confirm that the following items are present in the figure legend, table legend, main text, or Methods section.

n/a	Confirmed	
☐	☒	The exact sample size (*n*) for each experimental group/condition, given as a discrete number and unit of measurement
☒	☐	A statement on whether measurements were taken from distinct samples or whether the same sample was measured repeatedly
☐	☒	The statistical test(s) used AND whether they are one- or two-sided<br>*Only common tests should be described solely by name; describe more complex techniques in the Methods section.*
☒	☐	A description of all covariates tested
☒	☐	A description of any assumptions or corrections, such as tests of normality and adjustment for multiple comparisons
☐	☒	A full description of the statistical parameters including central tendency (e.g. means) or other basic estimates (e.g. regression coefficient) AND variation (e.g. standard deviation) or associated estimates of uncertainty (e.g. confidence intervals)
☐	☒	For null hypothesis testing, the test statistic (e.g. *F*, *t*, *r*) with confidence intervals, effect sizes, degrees of freedom and *P* value noted<br>*Give P values as exact values whenever suitable.*
☐	☒	For Bayesian analysis, information on the choice of priors and Markov chain Monte Carlo settings
☒	☐	For hierarchical and complex designs, identification of the appropriate level for tests and full reporting of outcomes
☐	☒	Estimates of effect sizes (e.g. Cohen's *d*, Pearson's *r*), indicating how they were calculated

Our web collection on statistics for biologists contains articles on many of the points above.

Software and code

Policy information about availability of computer code

Data collection	No software was used.
Data analysis	Short-read data was analyzed using a pipeline described here: https://gitlab.com/tbgenomicsunit/ThePipeline with parameters adjusted described in the manuscript. Programs: FastP, Kraken (v0.10.5-beta), BWA (v0.7.10-r789), Samtools (v1.15), VarScan (v2.3.7), GATK HaplotypeCaller (v3.8), Python (v2.7.5), SnpEff (v4.1), snp-sites (v2.5.1) and PopArt Software (v1.7). Long-read data and complete genomes were analyzed with: LongQC (v1.2.0b), Kraken (v0.10.5), KrakenTools (v1.2), HifiAdapter (v2.0.0), minimap2 (v2.26), Python (v3.7), Flye (v2.9.2), Circlator (v1.5.5), pbmm2 (v1.13), Artemis (v18.2.0), vt (v0.57721), liftoff (v1.6.3), blastn (v2.12.0+), R (v4.2.2), freebayes (v1.3.6), BUSCO (v5.5.0), IDEEL, Merqury (v1.3), Prodigal (v2.6.3), QUAST (v.5.2.0), Sniffles2 (v2.3.3), Minigraph-Cactus (v2.8.4), HAL tools (v2.3), maf2synteny (v1.2), nucmer (v4.0.0rc1), paftools (v2.26), IQ-TREE2 (v2.2.5), TreeTime (v0.11.4), BEAST (v2.7.7), Tracer (v1.6), Variscan (v2.0.5), MACSE (v2.07), RDP5, Aliview (v.2021), Mesquite (v.4.02), FastaCon, javarkit, SNAP (v2.1.1), Nucdiff (v2.0.3), and progressiveMauve (v2.4.0). All original code has been deposited at Zenodo with DOI: http://doi.org/10.5281/zenodo.15489014 and is publicly available at https://github.com/anamatgu/MTBC_complete_genomes.

For manuscripts utilizing custom algorithms or software that are central to the research but not yet described in published literature, software must be made available to editors and reviewers. We strongly encourage code deposition in a community repository (e.g. GitHub). See the Nature Portfolio guidelines for submitting code & software for further information.

Data

Policy information about availability of data

All manuscripts must include a data availability statement. This statement should provide the following information, where applicable:

- Accession codes, unique identifiers, or web links for publicly available datasets
- A description of any restrictions on data availability
- For clinical datasets or third party data, please ensure that the statement adheres to our policy

All sequence data have been deposited on the European Nucleotide Archive in the projects PRJEB29604, PRJEB38719, PRJEB65844, PRJEB89456, PRJEB89397, PRJEB70424, and PRJEB89421 and are publicly available. The accession numbers for each sample are included in Supplementary Data 1, 2, and 10. Source Data are provided with this paper.

Research involving human participants, their data, or biological material

Policy information about studies with human participants or human data. See also policy information about sex, gender (identity/presentation), and sexual orientation and race, ethnicity and racism.

Reporting on sex and gender	We only analyzed bacterial genomic data. This is no applicable to our study because not human data is analyzed. Biological samples were collected without discrimination based on sex or gender.
Reporting on race, ethnicity, or other socially relevant groupings	We only analyzed bacterial genomic data. This is no applicable to our study because not human data is analyzed.
Population characteristics	Samples were collected in the Valencia Region, Spain, a low burden TB setting.
Recruitment	All culture-positive MTB cases were included in the study.
Ethics oversight	Ethics Committee for Clinical Research from the Valencia Regional Public Health Agency (Comité Ético de Investigación Clínica de la Dirección General de Salud Pública y Centro Superior de Investigación en Salud Pública, Reference 20210430/03)

Note that full information on the approval of the study protocol must also be provided in the manuscript.

Field-specific reporting

Please select the one below that is the best fit for your research. If you are not sure, read the appropriate sections before making your selection.

☒ Life sciences ☐ Behavioural & social sciences ☐ Ecological, evolutionary & environmental sciences

For a reference copy of the document with all sections, see nature.com/documents/nr-reporting-summary-flat.pdf

Life sciences study design

All studies must disclose on these points even when the disclosure is negative.

Sample size	This is a population-based genomic study capturing all TB cases in the Valencia Region, and therefore, not size sample was measured.
Data exclusions	This analysis is focused on sequencing Mycobacterium tuberculosis cultures. Therefore, only negative-culture TB cases were not elegible for the analysis.
Replication	Because our study was designed to compare different sequencing technologies using Mycobacterium tuberculosis samples, we did not use specific experimental groups. Instead, we verified the reproducibility of our findings through this cross-platform comparison and strict internal quality controls. By analyzing the same samples with both Illumina (short-read) and PacBio (long-read) technologies, we were able to evaluate the consistency of our results across different methods. All attempts to reconcile the data between the two platforms were successful, and our reported findings remained consistent across both the Illumina and PacBio datasets. To ensure the work is reproducible by others, we used peer-reviewed pipelines with standardized parameters and tools and have made all custom code publicly available. Furthermore, every assembly in our final analysis met high-quality thresholds. No findings were excluded due to a lack of reproducibility.
Randomization	This is not relevant for our study. There are not experimental groups in this study.
Blinding	This is not relevant for our study. There are not experimental groups in this study.

Reporting for specific materials, systems and methods

We require information from authors about some types of materials, experimental systems and methods used in many studies. Here, indicate whether each material, system or method listed is relevant to your study. If you are not sure if a list item applies to your research, read the appropriate section before selecting a response.

Materials & experimental systems

n/a	Involved in the study
☒	☐ Antibodies
☒	☐ Eukaryotic cell lines
☒	☐ Palaeontology and archaeology
☒	☐ Animals and other organisms
☒	☐ Clinical data
☒	☐ Dual use research of concern
☒	☐ Plants

Methods

n/a	Involved in the study
☒	☐ ChIP-seq
☒	☐ Flow cytometry
☒	☐ MRI-based neuroimaging

Plants

Seed stocks

Report on the source of all seed stocks or other plant material used. If applicable, state the seed stock centre and catalogue number. If plant specimens were collected from the field, describe the collection location, date and sampling procedures.

Novel plant genotypes

Describe the methods by which all novel plant genotypes were produced. This includes those generated by transgenic approaches, gene editing, chemical/radiation-based mutagenesis and hybridization. For transgenic lines, describe the transformation method, the number of independent lines analyzed and the generation upon which experiments were performed. For gene-edited lines, describe the editor used, the endogenous sequence targeted for editing, the targeting guide RNA sequence (if applicable) and how the editor was applied.

Authentication

Describe any authentication procedures for each seed stock used or novel genotype generated. Describe any experiments used to assess the effect of a mutation and, where applicable, how potential secondary effects (e.g. second site T-DNA insertions, mosiacism, off-target gene editing) were examined.
