# Supplementary material for: Complete genomes reveal a refined map of Mycobacterium tuberculosis genetic diversity across evolutionary scales
Source: Nat Commun. 2026 Jun 6;17:7242. doi: 10.1038/s41467-026-73869-5 (PMC13396471; doi:10.1038/s41467-026-73869-5)
Supplement: Supplementary file 14 — Transparent Peer Review file [file 41467_2026_73869_MOESM14_ESM.pdf]

nature portfolio

Peer Review File

Complete genomes reveal a refined map of *Mycobacterium tuberculosis* genetic diversity across evolutionary scales

Corresponding Author: Dr Iñaki Comas

This file contains all reviewer reports in order by version, followed by all author rebuttals in order by version.

Version 0:

Reviewer comments:

Reviewer #1

(Remarks to the Author)
Summary
This project used a combination of long-reads and short reads to study within-host heterogeneity, structural variation, gene conversion in PE/PPE loci, and T-cell epitope conservation in clinical MTBC isolates. While the datasets and assemblies are valuable, several core claims and the scope of the manuscript (as described by the authors) are not supported by the results. Significant lack of rigor in methods makes the discoveries uncertain and their evaluation not possible.
Major comments
1. Scope and title misalignment (Lines 1–2).
The title implies MTBC-wide coverage, but the data are overwhelmingly M. tuberculosis Lineage 4 (209 isolates) with minimal representation of other lineages/species (L2: 4, L3: 2; no L1 or L5–10; one M. bovis; none of M. africanum, M. canettii). I recommend reframing the study as one of diversity of L4, treating non-L4 isolates as outgroups. Given extensive prior work on L4 diversity, the incremental significance is limited.

2. Within-patient heterogeneity claims rely on short reads while excluding informative HiFi variation (Lines 33–34, 172–174, 355–357, 584–591).
The manuscript concludes M. tuberculosis harbors less heterogeneity than previously thought, yet heterogeneous calls from HiFi reads are excluded. The authors used short reads (mapped to HiFi-derived consensus references) to draw this conclusion while also reporting that short reads miss ~303 variants/sample. As their own data concludes, this strategy can under-estimate heterogeneity, especially in repetitive/PE/PPE regions masked or error-prone for short reads. This is particularly problematic as one of the major claims of this study is that M. tuberculosis has less heterogeneity than previously thought

3. Structural variation (SV) under-called and under-quantified (Lines 70–72, 151–152, 450).
The claim that SV contributes less to diversity than gene conversion is unsupported without systematic SV calling. A tool designed for SV detection should be run on all samples; report genome-wide and PE/PPE-focused SV counts, size spectra, allele frequencies, and functional context. Current manual BLAST-based inspection focusing on IS6110 and ppe38-71 is insufficient to support global conclusions.

4. Gene conversion detection and validation (Lines 257–260).
Gene conversion is notoriously prone to false positives, particularly in PE/PPE. Methods severely lack rigor. Events need to be corroborated using orthogonal methods. Stringent filters need to be implemented, especially for controlling the FDR. Finally, explicitly distinguish gene conversion from tandem duplication or segmental duplication.

5. Annotation strategy limits discovery of novel gene content (Lines 131–133).
Liftoff against H37Rv will miss lineage- or isolate-specific genes absent in the reference. To capitalize on complete genomes, complement with a de novo/pan-genome workflow (e.g., isolate-level annotation plus clustering across genomes) and report novel genes/pseudogenes and their distribution. Using CDC1551 for ppe38-71 is a useful fix; a broader approach is still needed.

6. Incomplete background research. In general, the manuscript suffers from incomplete review of existing literature. As a result, in some parts, the significance of aspects of this work is overstated, methods incorrectly applied, or incorrect

conclusions are reached.

Minor comments
• Priors rationale (Lines 223, 388–391). It is not explained how these specific three mean SD pairs were chosen as prior scenarios.
• Conversion vs duplication (Lines 257–260). The procedure described would also find gene duplication events. How were these distinguished from gene conversion events?
• BLAST thresholds (Lines 257–260). No query thresholds are described for detecting conversion events.
• Section naming (Lines 299–300). It is at first unclear how this methods subsection is distinct from the transmission network reconstruction from short reads procedure described in lines 189-192. To emphasize the distinction, add “with complete genomes” or “using long reads” to this subsection’s title and opening sentence.
• PE/PPE virulence claims (Line 453). There is disagreement on the effects of ppe38/ppe71 disruption; contextualize with recent and legacy literature (e.g., Koleske et al. 2025; Ates et al. 2018; Dong et al. 2012; Weerdenburg et al. 2012).
• Epitope diversity (Lines 502–520). Since T-cells are not the only immune response, the significance of T-cell epitope conservation results is overstated without a comprehensive immune response assessment.

(Remarks on code availability)
Code was not evaluated

Reviewer #2

(Remarks to the Author)
This very interesting and important manuscript by García-Marín et al provides a relevant framework for the integration of complete genome sequences not only in transmission and evolution analysis but it also lays some groundwork towards the study of pathogen adaptation and genome plasticity. Moreover, the article is very well written, and the methods fully conveys the complexity of the analytical framework, the QC evaluation and analysis is well defined and the complete set of bioinformatic tools used is impressive and denotes a careful and thorough analysis of the data that supports the findings and conclusions. My comments are mainly directed at improving the manuscript and the clarity of some of the main findings.
One of main limitations relates to the fact that the set of clinical isolates used in this study lacks epidemiological data, which hampers the integration of information concerning epidemiologically linked cases and cases associated with cryptic transmission events into the differently inferred transmission networks. This can be mentioned as a limitation, highlighting the need to investigate further.
In the Introduction, the authors appropriately highlight the importance of diverse regions of the genome and its potential for within-host studies. Perhaps the authors could add 2-3 lines on the importance of within host evolution to the identification and understanding of the role played by key genes in the adaptation to the host immune response.
The MTBC Ancestor used for alignment was inferred back in the seminal work by Comas et al in 2010. If I recall correctly, it was obtained from the consensus upon mapping short read data? Here, the authors are using long-read data to establish reference genomes but are departing from one obtained using short-read data. Are there any possible consequences? Please discuss.
The authors used Kraken to undertake read taxonomical classification and excluded reads based on this analysis. Please clarify why wasn’t kraken2 used, which Kraken database was used and how this might affect the results? Was it necessary to remove these reads? Was this also done for short-read data?
Why did the authors opted for a strict molecular clock in BEAST when the rate can vary across branches? Did the authors attempted a different approach using other molecular clock models?

RESULTS

The evolutionary rate observed when considering complete genomes is about 1.5 fold higher. Do the authors credit this to the highly variable regions excluded? When performing the ascertainment bias correction in BEAST did authors used different genome lengths, and hence different number of invariable sites in short read data since regions difficult to map were excluded? I am asking because when using sequences with higher number of variants without correcting for this it might, by itself, lead to higher substitution rate estimates.

Line 455 – The authors mention that some regions were completely absent. Could this be confirmed by PCR or other method? To confirm that this is not a sequencing/assembly issue.

Would it be possible for the authors to describe if there is any clear association of gene conversion events and the phylogenetic structure of the tree, i.e., association with specific branches or subtrees and when did these might have occurred? Or, if there is evidence of independent occurrences of similar gene conversion events? There is a tree depicting the distribution, but it is not well addressed in the text.
Figure 7 – It is interesting to observe that some pairs of isolates distancing 1 SNP when using short-read data end up at 15/16 SNPs apart when using complete genomes. Looking at the increase generally observed for the rest of the data, the magnitude of this increase is unusual. What does account for this, and can the authors offer an explanation?
Concerning the discussion a popping question is: how does the integration of additional resolution in transmission scenarios help with our understanding of transmission and our capacity to detect and act upon. This is something the authors aim at but a clear vision for this integration or its need is not discussed or put into a broader perspective.

Minor Issues:
Line 75-77 – at this stage, this sentence may be confusing as no background was given before.
Lines 186 – The authors mention the WHO mutation catalogue as a basis for identifying resistance associated variants. Yet some mutations, owing to its low frequency or association with specific clades, may still be classified as mutations with uncertain association. How was this dealt with?
Lines 272-275 – did any type of epitope sequence clustering was done at IEDB to remove redundancy between epitope records. It is possible that there is overlap between sequence entries that may relate to the same epitope.
Line 285 – Please clarify how this dN/dS ratio was obtained. Is it the medians from pairwise comparisons in an alignment? How did you ensure a codon-aware alignment at this scale of genomes and genes?
Figure 2- Please explain better in the legend that the distribution by COGs is just for the genes associated with mutation hotspots, correct?

(Remarks on code availability)

Version 1:

Reviewer comments:

Reviewer #1

(Remarks to the Author)
Key results
This resubmission presents a large set of PacBio HiFi complete genomes from clinical isolates in Valencia and uses those assemblies to argue that complete genomes recover substantial additional variation beyond standard short-read analyses, identify pe/ppe diversity hotspots shaped by gene conversion, improve transmission resolution, and revise current views of within-host diversity. The dataset is potentially valuable. However, after revision, I remain unconvinced that the manuscript supports its current title, abstract, and central framing. The paper still presents a largely Lineage-4 regional dataset as a general map of Mycobacterium tuberculosis genetic diversity across evolutionary scales. That mismatch between dataset and claim remains the principal obstacle to publication.
Validity
1. The main scope problem remains unresolved. The manuscript still makes species-level claims about Mycobacterium tuberculosis genetic diversity, yet the study population is a regional Valencia cohort overwhelmingly dominated by Lineage 4. Adding statements that the dataset is “mostly” or “enriched in” Lineage 4 does not solve the problem when the title, abstract, and conclusions continue to generalize to M. tuberculosis as a whole.
2. The within-host conclusions remain broader than the data support. This part of the study analyzes serial short-read isolates from only five patients, mapped to patient-specific baseline complete genomes. That design can support a narrower conclusion about the effect of reference choice on false-positive non-fixed variant calls, but it does not justify a broad reassessment of within-host diversity in M. tuberculosis.
3. The structural-variation component is still not presented as a systematic genome-wide analysis. Selected findings at ppe38-71 and limited epitope-focused observations may be interesting, but they do not support broad statements about the structural-variation landscape or its overall contribution to diversity.
4. The gene-conversion section is improved relative to the original submission and is no longer my main concern. The revised methods are clearer and the orthogonal validation is a genuine improvement. Even so, that technical progress does not overcome the central framing problem described above.

Significance
Because the dataset is overwhelmingly Lineage 4 from one geographic setting, the current manuscript does not support the breadth of significance claimed for a general-interest journal audience. The work would be more compelling and more credible if it were framed as a focused study of a Lineage-4-enriched population-based complete-genome dataset and its implications for repetitive-region diversity, reference choice, and selected local epidemiologic inferences. In its current form, the manuscript overstates the generality of the conclusions.

Data and methodology
5. The complete-genome dataset itself appears valuable and the manuscript is stronger than the original version in describing how gene-conversion candidates were identified and cross-checked.
6. However, the annotation strategy remains limited for discovery of lineage- or isolate-specific gene content. A lift-over strategy anchored to H37Rv is practical, but it does not provide a true pangenomic view and therefore cannot support broad conclusions about novel gene content or gene-content evolution.
7. For structural variation, the current analyses remain selective rather than comprehensive. If the authors wish to retain stronger SV claims, they need a systematic genome-wide calling framework and a quantitative summary of events, sizes, frequencies, and affected loci.

Analytical approach
8. Key quantitative analyses are effectively Lineage-4 analyses. That is acceptable for the dataset at hand, but it reinforces the need to narrow the manuscript-wide claims.
9. The comparison between universal-reference and patient-specific-reference pipelines is informative, but the interpretation

should be restricted to what was actually tested: false-positive behavior in short-read calling, not the full extent of within-host diversity accessible with complete genomes.
10. The revised analyses are technically improved, but the inferential reach of the manuscript still exceeds the reach of the underlying study design.

Clarity and context
The manuscript is clearer than the previous version, but the central message remains internally inconsistent: the authors acknowledge the Lineage-4 enrichment and then continue to frame the study in organism-wide terms. That tension should be resolved directly. There are also residual textual issues that suggest the paper still needs careful editorial polishing, including duplicated phrasing and a few wording or figure-reference problems.

References
The literature context is improved, but some of the broad interpretive statements still outpace the evidence presented. The manuscript would benefit from more explicit positioning relative to prior work on Lineage 4, MTBC pangenome diversity, and structural variation.

(Remarks on code availability)
My assessment covers bacterial genomics, comparative genomics, tuberculosis genomics, variant calling, and evolutionary interpretation. I did not independently verify every computational intermediate, but the main concerns raised above are conceptual and inferential rather than dependent on re-running the full pipeline.

Reviewer #2

(Remarks to the Author)
This revised version incorporates several changes that have clarified some aspects that were not entirely clear. Furthermore, the authors answered all my questions and addressed the concerns that I have raised by undertaking further data analysis and integrating the results in the main text for discussion and analysis. I have some additional and minor comments:

As an optional point for discussion - the authors determined an increased mutation rate upon including full genome sequence data. Should this type of sequence data be analysed at the light of less stringent cutoffs?

In the attempt to respond to the concerns raised by Reviewer 1, the authors have changed the title to include the term “evolutionary scales” to characterize the scope of the sample and the breadth of conclusions that can be drawn. Personally, I find this term poorly informative and quite vague. I do agree that the overall genetic diversity of this sample is somewhat limited, but this can be discussed and acknowledged as a limitation.

L301-304 – The authors refer to annotation by lift over “as previously described” yet provide no reference.

(Remarks on code availability)

Open Access This Peer Review File is licensed under a Creative Commons Attribution 4.0 International License, which permits use, sharing, adaptation, distribution and reproduction in any medium or format, as long as you give appropriate credit to the original author(s) and the source, provide a link to the Creative Commons license, and indicate if changes were made.
In cases where reviewers are anonymous, credit should be given to 'Anonymous Referee' and the source.
The images or other third party material in this Peer Review File are included in the article's Creative Commons license, unless indicated otherwise in a credit line to the material. If material is not included in the article's Creative Commons license and your intended use is not permitted by statutory regulation or exceeds the permitted use, you will need to obtain permission directly from the copyright holder.
To view a copy of this license, visit https://creativecommons.org/licenses/by/4.0/

Reviewer 1

Summary

This project used a combination of long reads and short reads to study within-host heterogeneity, structural variation, gene conversion in PE/PPE loci, and T-cell epitope conservation in clinical MTBC isolates. While the datasets and assemblies are valuable, several core claims and the scope of the manuscript (as described by the authors) are not supported by the results. Significant lack of rigor in methods makes the discoveries uncertain and their evaluation not possible.

We appreciate the reviewer's criticisms, although we don't agree with the considerations. Particularly, we think novelty is a matter of discussion while analysis rigor is not. And, in this regard, we believe that the amount of work presented and the approaches employed show that we have followed the most appropriate analyses, as also recognized by Reviewer 2. In any case, there are points raised by reviewer 1 that we think are valid, and we have tried to address them below.

Major comments

R1.1. Scope and title misalignment (Lines 1–2). The title implies MTBC-wide coverage, but the data are overwhelmingly M. tuberculosis Lineage 4 (209 isolates) with minimal representation of other lineages/species (L2: 4, L3: 2; no L1 or L5–10; one M. bovis; none of M. africanum, M. canettii). I recommend reframing the study as one of diversity of L4, treating non-L4 isolates as outgroups. Given extensive prior work on L4 diversity, the incremental significance is limited.

In fact, we explicitly recognize from the very beginning that our dataset is enriched in Lineage 4 isolates (both in Introduction and Discussion). This corresponds to the prevalence of M. tuberculosis in our region, similar to the whole country, and we must stress the need to work with samples from our area because we wanted to analyze diversity at multiple levels from the same dataset. This continuum cannot be achieved otherwise.

Still, we agree that most diversity is contributed by lineage 4. Because of its relevance, it has been the focus of many previous studies with short reads (Stucki et al., 2016), as mentioned by the reviewer. But we don't agree that there is "extensive prior work" on L4 and long reads/complete genomes. Ours is the first analysis with this breadth, which allows us to describe phenomena not described before (genetic diversity hotspots, entire *pe/ppe* gene repertoire, extent of gene conversion in *pe/ppe* genes, evolutionary rate estimates, etc.). None of these have been described to this extent before because there was no dataset of complete genomes of this quality available before.

However, to avoid any potential ambiguity regarding scope and the high presence of Lineage 4 in our dataset, we have clarified it in the Abstract/Introduction and also identified genetic diversity as the main goal in the title.

New title:
Complete genomes reveal a refined map of Mycobacterium tuberculosis genetic diversity across evolutionary scales.

Abstract:
Lines 19-35

Introduction:
Lines 76-87: *The global analysis of this dataset, enriched in Lineage 4, reveals that complete genomes capture substantially more genetic diversity than the matched short-read data. Most genetic diversity hotspots map to the pe/ppe gene family, a largely uncharacterized group in mycobacteria, with gene conversion emerging as the main driver of nucleotide diversity in these regions. Despite the high overall variability of pe/ppe genes, epitopes from PE/PPE antigens are hyperconversed, with notable exceptions included in vaccine candidates. At the epidemiological scale, incorporating SNPs from overlooked regions, indels, and structural variants provides higher resolution for defining genetic transmission boundaries and networks. Finally, we use a complete genome from the same patient as a reference to map short reads from serial isolates to reliably detect genuine within-host variation. This strategy reveals a substantial reduction in non-fixed variation due to false positives introduced by conventional pipelines using a global reference, prompting a reassessment of current within-host diversity estimates and the dynamics of M. tuberculosis during infection.*

R1.2. Within-patient heterogeneity claims rely on short reads while excluding informative HiFi variation (Lines 33–34, 172–174, 355–357, 584–591). The manuscript concludes M. tuberculosis harbors less heterogeneity than previously thought, yet heterogeneous calls from HiFi reads are excluded. The authors used short reads (mapped to HiFi-derived consensus references) to draw this conclusion while also reporting that short reads miss ~303 variants/sample. As their own data concludes, this strategy can under-estimate heterogeneity, especially in repetitive/PE/PPE regions masked or error-prone for short reads. This is particularly problematic as one of the major claims of this study is that M. tuberculosis has less heterogeneity than previously thought.

We apologize for not being clear in the text, which likely led the reviewer to misinterpret our results. We use the dataset of complete genomes to analyse genetic diversity at three scales: global, epidemiological, and within-host. The reviewer raises two different questions:

First, why is the large variation observed between patients not observed in the within-patient analysis?

The reviewer mentions the 303 median number of SNPs fixed between the two samples compared (being fixed those mutations with allele frequency in the culture >90%), but the range is wide depending on how much time separates the two strains analyzed. When looking at diversity within a patient, we talk about weeks of difference, and thus, the median number of changes between samples of the same patient is usually 0. In turn, what is observed is a large number of mutations with allele frequency below 90%, revealing diversification within the host but not yet fixed in the lung population. Thus, the number of

differences at the population scale cannot be directly compared to the number of differences within a patient, as the nature of the diversity analyzed is different. This has been shown by many publications (Vargas et al., 2021, for example).

Also, the reviewer may be confused by the fact that when we talk about allele frequencies, we refer to the frequency of a mutation in bulk sequencing of the culture of a TB case, while in population genetics, allele frequencies are often used when talking about the site-frequency mutation spectra at a population level. We have tried to remark on the meaning of allele frequency in our paper, which follows the standard in the TB field.

The second question is related to those regions that are now available for analysis, thanks to using complete genomes, but were excluded in the within-host analysis when using short-read sequencing.

We did not generate complete genomes for all serial isolates but only for the baseline sample of each group (transmission cluster or patient). For the within-host analyses, we mapped short reads to the corresponding baseline complete genome. We did not attempt to analyze those informative regions because they are still inaccessible by short-read data. We aimed at generating a clean comparison between regions analyzed when using a conventional short-read reference mapping pipeline compared to using a patient- or cluster-specific reference.

Thus, the goal of this analysis is to show that the detection of within-host diversity from short-read sequences is more accurate when mapping to a patient-specific reference genome than to a universal reference genome.

We have tried to clarify these points in the text by adding a paragraph in the introduction, as suggested by Reviewer #2, and changing the wording of some sentences:

Lines 53-61: *Another common limitation of reference-mapping approaches is the use of a single reference genome to analyse strains across all MTB lineages, typically H37Rv or an H37Rv ancestral-like sequence that preserves genome architecture while incorporating ancestral alleles. Although the MTB is often described as monomorphic, reference choice can influence genetic diversity estimates (Valiente-Mullor et al. 2021; Harrison et al. 2024; Lee et al. 2016). This issue is particularly relevant for the detection of within-host variation, where non-fixed variants are prone to false-positive calls, challenging reliable inference (Martin et al. 2018). Accurate identification of within-host variation is critical, as it can reveal heteroresistance and selective processes during host–pathogen interactions, and can be used to increase the resolution of genomic transmission networks, as shown by Lee et al. (Lee et al. 2020).*

Lines 83-87: *Finally, we use a complete genome from the same patient as a reference to map short reads from serial isolates to reliably detect genuine within-host variation. This strategy reveals a substantial reduction in non-fixed variation due to false positives introduced by conventional pipelines using a global reference, prompting a reassessment of current within-host diversity estimates and the dynamics of M. tuberculosis during infection.*

Lines 96-98: *Only one representative sample from each patient was included in all the analyses except for the within-host diversity analysis, in which we analyzed serial short-read sequences from five patients (see below).*

Lines 186-189: *Additionally, positions with two or more alleles at frequencies between 0.1 and 0.89 in each complete genome were individually masked in the MGA, ensuring that only fixed variants were included in the downstream analyses, defined here as mutations with an allele frequency >0.9 in the culture of each MTB isolate.*

R1.3. Structural variation (SV) under-called and under-quantified (Lines 70–72, 151–152, 450). The claim that SV contributes less to diversity than gene conversion is unsupported without systematic SV calling. A tool designed for SV detection should be run on all samples; report genome-wide and PE/PPE-focused SV counts, size spectra, allele frequencies, and functional context. Current manual BLAST-based inspection focusing on IS6110 and ppe38-71 is insufficient to support global conclusions.

We believe the reviewer may have been misled by a sentence that was not clear from our side in the process of reducing the number of words/synthesizing:

Misleading lines 71, 72, 73 (old manuscript): *Point mutations and structural variations contribute to that diversity, but it is mainly driven by gene conversion events, contrary to what is observed in the rest of the genome.*

In fact, we wanted to emphasize that gene conversion has not been described/reported in MTBC outside certain *pe/ppe* genes (and certain *esx* genes), separating *pe/ppe* from the rest of the genome. In addition, in our manuscript, structural variation was screened systematically only for locus *ppe38_71.* This locus is particularly interesting because it includes one of the most variable regions of the MTB genome and is likely linked to virulence. We apologize as the above sentence didn't make it clear, and we have removed it.

We have not looked at SV across PE/PPE because this is not the focus of this publication; we are focusing mainly on point mutations and gene conversion. While we are working on other aspects of genome variation (gene content, SV…etc), we think that it deserves another publication as the present ms is extensive enough with the analyses presented. We only included a comment regarding structural variants in PE/PPE epitopes to reinforce the idea that they are particularly converse. To make this clearer in the text, we have also reformulated this line and added one paragraph about the methodology to detect SVs:

Lines 344-347: *Finally, to detect indels and structural variants in PE/PPE epitopes, we identified gaps in the epitope MSAs, either in the MTBCA reference (indicative of insertion-like variants) or in the complete genomes (indicative of deletion-like variants). Putative variants were then classified using Nucdiff with default parameters by aligning the MTBCA reference to the corresponding complete genome.*

Lines 591-593: *Regarding indels and structural variation in PE/PPE epitopes, we detected a small deletion (6 bp) affecting one epitope of ppe18 in two isolates, and a large deletion (4670 bp) affecting one epitope of pe_pgrs35 in one isolate.*

R1.4. Gene conversion detection and validation (Lines 257–260).
Gene conversion is notoriously prone to false positives, particularly in PE/PPE. Methods severely lack rigor. Events need to be corroborated using orthogonal methods. Stringent filters need to be implemented, especially for controlling the FDR. Finally, explicitly distinguish gene conversion from tandem duplication or segmental duplication.

We partially agree with the reviewer. Gene conversion methods are usually based on homologous recombination detection approaches; they tend to overcall events, as many are based on the accumulation of mutations in short tracks, but cannot be confirmed because of the absence of matching mates in other regions of the genome. In that regard, we disagree about the lack of rigour for two reasons:

First, we have followed a methodology already published (Stritt et al., 2025), which is especially suitable for MTBC. This is because diversity in MTBC is extremely low, even in some regions with gene conversion, making most published approaches unsuitable. We agree that some methodologies yield false positives, this is why we regard the GUBBINS analysis as preliminary. GUBBINS looks for regions of mutation accumulation, making no assumptions about the mechanism(s) behind that accumulation. We may have failed to convey the preliminary status of the GUBBINS results (although mentioned in the original draft). In fact, we use blast and manual inspection to identify if the regions suspected to undergo gene conversion are confirmed, and discard other processes mentioned by the reviewer, such as tandem duplications or segmental duplications. In that sense, our approach is the most sensitive to discard the phenomena the reviewer is mentioning. The differentiation between gene conversion and duplications is addressed in detail in comment R1.8.

Second, the detection of gene conversion is usually carried out by tools designed to identify recombination. However, recombination and gene conversion have subtle differences, making those tools of limited use in differentiating between the two processes. For example, in recombination analysis, the parental strains are usually present in the alignment, but this is usually not the case for gene conversion, in which donors are usually in paralogous regions not included in the alignment under consideration. This limits alternative approaches to the one we have used. Still, to confirm our BLAST+ manually inspected events, we have run the RDP5 software and retained all recombinant regions confirmed by the GENECONV tool or other ones included in the software, thus providing orthogonal evidence for gene conversion as requested by the reviewer. Only events in one gene were not confirmed, but inspection of the alignment clearly points to gene conversion. Shown are two screenshots from the multiple sequence alignment of *pe_pgrs52*. The first shows a cluster of SNPs detected in three samples relative to the MTBCA reference (coordinates: 1340–1459). The second shows the adjacent region (coordinates: 1577–1688), highlighted in grey, which corresponds to the putative donor sequence (100% sequence identity).

Gene conversion event:

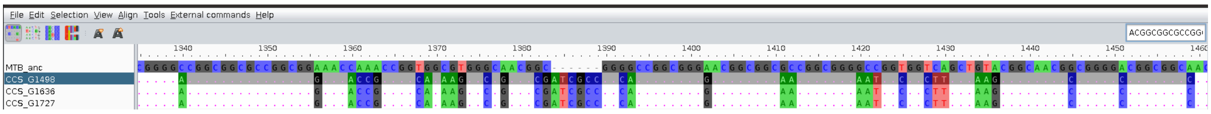

Donor sequence:

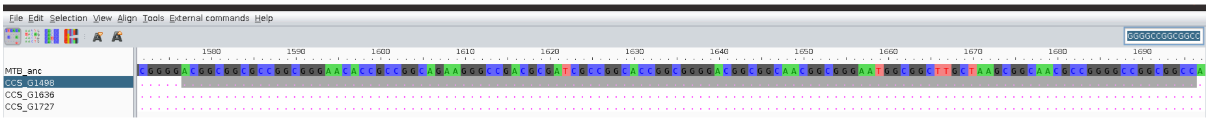

To clarify the previous and new methodology, we included a new section in methods (lines 293-321). Additionally, we provide the new results in the results section (lines 511-525). We have generated a new figure (Fig. 3c, Line 534) to replace the figure from GUBBINS, where we only represent confirmed gene conversion events. Further analyses of gene conversion are presented as part of the response to Reviewer 2.

R1.5. Annotation strategy limits discovery of novel gene content (Lines 131–133).
Liftoff against H37Rv will miss lineage- or isolate-specific genes absent in the reference. To capitalize on complete genomes, complement with a de novo/pan-genome workflow (e.g., isolate-level annotation plus clustering across genomes) and report novel genes/pseudogenes and their distribution. Using CDC1551 for ppe38-71 is a useful fix; a broader approach is still needed.

We appreciate this suggestion and agree that *de novo* annotation is a logical next step for our data. The present analysis is focused on genetic diversity, and that is now better reflected in the abstract and title. We are now working on identifying novel genetic elements as suggested by the reviewer. But this is entirely a new publication as it requires a vast amount of work and curation, as shown by recent publications of the MTBC pangenome (Behruznia et al., 2025) and the impact of structural variation (Calanda-Baltrons et al., 2025).

R1.6. Incomplete background research. In general, the manuscript suffers from incomplete review of existing literature. As a result, in some parts, the significance of aspects of this work is overstated, methods incorrectly applied, or incorrect conclusions are reached.

We appreciate the reviewer's comment, but our manuscript already has 99 citations. We think most relevant studies have been cited, but in any case, we would be happy to add key citations suggested by the reviewer. We have included some citations about the *ppe38_71* locus suggested in the minor comments. In addition, we have toned down or better contextualized some sentences in the discussion to clarify our conclusions.

Minor comments
R1.7. Priors rationale (Lines 223, 388–391). It is not explained how these specific three mean SD pairs were chosen as prior scenarios.

We thank the reviewer for raising this point, and we agree that the rationale for the three prior scenarios (tMRCA) should be explained more explicitly. Our goal in this section was not

to estimate the molecular clock rate of *M. tuberculosis* per se, but to compare rate estimates across different genome reconstructions (short-read mapping excluding repetitive regions vs. long-read assemblies including the complete genome).

Because our dataset is homochronous (all modern isolates were sampled in 2016), the temporal information available for tip-dating is limited, which can hinder clock calibration (Menardo et al., 2019). To mitigate this limitation and to enable a fair comparison between genome representations, we defined a set of tree height (tMRCA) priors for the complete-genome (PacBio) BEAST analysis, derived from the short-read (Illumina) analysis.

Strategy to define the tree height priors (root age priors).

1. **Short-read analysis to obtain an empirical calibration**. To introduce a temporal range, we included the ancient MTB genome dated to the 17th century ("bishop" genome; Salin et al., 2020) in the short-read-based analysis. This allowed us to estimate the posterior distribution of the tree height (tMRCA) for the full dataset.

2. **Using the short-read posterior as a prior for the complete genomes analysis.** The ancient genome is not available as long-read data and therefore cannot be included in the complete genomes dataset. To provide temporal information for the complete genomes analysis while keeping the design comparable, we used the Illumina posterior tree height (tMRCA) as an informative tree height prior in BEAST.

3. **Rationale for the three mean–SD pairs.** The three Normal prior scenarios were chosen to cover increasing levels of uncertainty around the short-read-derived tMRCA posterior, and to test the robustness of our analyses to prior specification:
Scenario i (mean = 200, SD = 150): a very diffuse prior deliberately allowing a wide plausible range (including and exceeding the short reads 95% HPD), representing a conservative/weakly-informative setting.
Scenario ii (mean = 300, SD = 100): an intermediate prior, centered close to the short reads central tendency but allowing substantially broader uncertainty.
Scenario iii (mean = 316, SD = 20): a tight prior centered on the short reads posterior median (restricted uncertainty; informative setting).

We have clarified the choice in the method section.

Lines 241-246: *modeled as a normal distribution under three scenarios: (i) a weakly informative diffuse prior (mean = 200, SD = 150) covering a broad range beyond the short-read 95% HPD; (ii) an intermediate prior (mean = 300, SD = 100) centered on the short-read data tendency with expanded variance; and (iii) an informative prior (mean = 316, SD = 20) constrained to the short-read posterior median. The three Normal prior scenarios were chosen to span increasing levels of uncertainty around the short-read-derived tMRCA posterior, and to test robustness to prior specification.*

R1.8. Conversion vs duplication (Lines 257–260). The procedure described would also find gene duplication events. How were these distinguished from gene conversion events?

To detect gene conversion events, we first obtained a Multiple Sequence Alignment for each individual *pe/ppe* gene, incorporating the inferred MTBC ancestor as the reference. We identified clusters of SNPs in each isolate relative to this reference. These candidate regions were then queried using BLAST against both the query's own complete genome and the MTBC ancestor reference.

When two 100%-identity hits were found within the query's complete genome, we identified the annotation of the corresponding regions through coordinate conversion with the reference. One hit corresponds to the original locus observed in the MSA, while the second identifies the putative donor gene. Because both sequences map to distinct, pre-existing annotated loci in the reference, we can rule out duplications. The presence of both regions in the reference genome confirms that the observed variation results from gene conversion between existing paralog regions rather than a duplication event.

We have reformulated this method section to clarify the approach.

Lines 301-304: *Both the targeted and the source regions are annotated by lift over as previously described, confirming that both are aligned against the reference genome, thus discarding the possibility of duplications. In the case of gene conversion within a gene, tandem and segmental duplications were discarded by the visualization of the MSA with Aliview (v.2021).*

R1.9. BLAST thresholds (Lines 257–260). No query thresholds are described for detecting conversion events.

We used an ID percentage of 100%. Thus, we are likely underestimating events, particularly when the region under gene conversion has diversified after the event.

R1.10. Section naming (Lines 299–300). It is at first unclear how this methods subsection is distinct from the transmission network reconstruction from short reads procedure described in lines 189-192. To emphasize the distinction, add "with complete genomes" or "using long reads" to this subsection's title and opening sentence.

As suggested by the reviewer, we have rephrased the subheader, and we have also clarified it in the text:

Line 358: *Multiple genome alignments to reconstruct transmission networks with complete genomes*

Line 359-361: *Individual MGAs were constructed using progressiveMauve (v2.4.0) with default parameters using complete genomes, for each genomic transmission cluster previously identified by short-read data, applying a 10-SNP threshold.*

R1.11. PE/PPE virulence claims (Line 453). There is disagreement on the effects of ppe38/ppe71 disruption; contextualize with recent and legacy literature (e.g., Koleske et al. 2025; Ates et al. 2018; Dong et al. 2012; Weerdenburg et al. 2012).

We have changed the wording and added two citations suggested by the reviewer.

Lines 527-530: *We were particularly interested in structural polymorphisms in the ppe38-71 locus, as it is one of the most variable regions of the MTB genome, and it has been linked to the secretion of virulence factors such as PE_PGRS and PPE-MPTR proteins (Ates 2020; Ates et al. 2018). Inactivation of this locus by deletion or truncation of ppe38 exhibits diverse effects on virulence in mouse models of infection (Ates 2020; Koleske et al. 2025).*

R1.12. Epitope diversity (Lines 502–520). Since T-cells are not the only immune response, the significance of T-cell epitope conservation results is overstated without a comprehensive immune response assessment.

We agree with the reviewer in that immune responses to TB are more complex than T-cell epitopes alone. However, it is known that T-cells are key components of protective adaptive immunity in early TB infection (Flynn et al. 2022)

However, in this case, we didn't want to discuss the biological implications but to reproduce an analysis (T-cell conservation) that was not possible for *pe/ppe* genes before the availability of complete genomes and compare it with previous data, thus restricting the range of antigens analyzed. We have introduced a sentence in the discussion to make this clear. Thanks for the suggestion.

Lines 734-736: *T-cell-mediated immunity is central for protective responses in TB (O'Garra et al. 2013; Flynn and Chan 2022), with previous studies showing conservation of T-Cell antigens in epitope regions (Comas et al. 2010; Coscolla et al. 2015).*

Reviewer #2 (Remarks to the Author):

This very interesting and important manuscript by García-Marín et al provides a relevant framework for the integration of complete genome sequences not only in transmission and evolution analysis, but it also lays some groundwork towards the study of pathogen adaptation and genome plasticity. Moreover, the article is very well written, and the methods fully conveys the complexity of the analytical framework, the QC evaluation and analysis is well defined and the complete set of bioinformatic tools used is impressive and denotes a careful and thorough analysis of the data that supports the findings and conclusions. My comments are mainly directed at improving the manuscript and the clarity of some of the main findings.

R2.1. One of main limitations relates to the fact that the set of clinical isolates used in this study lacks epidemiological data, which hampers the integration of information concerning epidemiologically linked cases and cases associated with cryptic transmission events into the differently inferred transmission networks. This can be mentioned as a limitation, highlighting the need to investigate further.

In fact, we have associated clinical data that were reported in a previous publication (Cancino-Muñoz et al., 2022). There, we already showed that many transmission events detected by genomic clustering are from community contacts not detected by routine contact tracing from Public Health Offices. In addition, in the present study, we only analyze one year of genomic data; the number of confirmed contacts by contact investigations is very low, precluding any analysis on how they relate to the new genetic distances calculated with complete genomes. Here, we focus on the additional information gained just by genetic information, always highlighting that genetic networks do not necessarily recapitulate transmission (as we discussed in Cancino-Muñoz et al., 2019). In agreement with the reviewer, we have added a comment in the discussion.

Discussion lines 769-771: *Although the limited number of confirmed contact pairs in our current dataset precludes definitive conclusions, larger benchmarking datasets spanning a broader timeframe will help formally test this trend.*

Isolate_ID	Isolate_ID	Detected by public health	Days	Illumina mapping	Complete	
G1498	G1499	yes	36	634	928	
G1544	G1630	yes	2	0	0	
G1693	G1661	yes	23	3	3	
G1653	G1785	yes	47	0	0	
G1537	G1786	yes	109	1	0	*False positive SNP in Illumina data
G1616	G1967	yes	29	721	1066	
G1604	G1704	yes	111	0	0	
G1704	G1821	yes	66	1	2	
G1604	G1821	yes	177	1	2	

R2.2. In the Introduction, the authors appropriately highlight the importance of diverse regions of the genome and its potential for within-host studies. Perhaps the authors could add 2-3 lines on the importance of within-host evolution to the identification and understanding of the role played by key genes in the adaptation to the host immune response.

We have now expanded our explanation in the Introduction about the importance of within-host variation:

Lines 53-61: *Another common limitation of reference-mapping approaches is the use of a single reference genome to analyse strains across all MTB lineages, typically H37Rv or an H37Rv ancestral-like sequence that preserves genome architecture while incorporating ancestral alleles. Although the MTB is often described as monomorphic, reference choice can influence genetic diversity estimates (Valiente-Mullor et al. 2021; Harrison et al. 2024;*

Lee et al. 2016). This issue is particularly relevant for the detection of within-host variation, where non-fixed variants are prone to false-positive calls, challenging reliable inference (Martin et al. 2018). Accurate identification of within-host variation is critical, as it can reveal heteroresistance and selective processes during host–pathogen interactions, and can be used to increase the resolution of genomic transmission networks, as shown by Lee et al. (Lee et al. 2020).

R2.3. The MTBC Ancestor used for alignment was inferred back in the seminal work by Comas et al in 2010. If I recall correctly, it was obtained from the consensus upon mapping short read data? Here, the authors are using long-read data to establish reference genomes but are departing from one obtained using short-read data. Are there any possible consequences? Please discuss.

The major difference is that our ancestor-reference is H37Rv-like in terms of gene content and structural variation. So the limitations are similar to when H37Rv is used as a reference genome. Any region missed by H37Rv will not be represented in mapping analyses. On the other hand, using H37RV-like reference means that we can incorporate the very rich information in terms of annotation and other biological insights from H37Rv. There have been attempts to use a new ancestral genome with all regions included (Harrison et al., 2024). We now mention in the text this possibility as well as highlight that our dataset will contribute to infer better reference genomes for mapping.

Discussion lines 781-783: *Even in the case where closer references are not available, our dataset provides a robust repository of high-quality complete genomes from several lineages and sub-lineages of L4 that allows the selection of optimal references for future analyses.*

R2.4. The authors used Kraken to undertake read taxonomical classification and excluded reads based on this analysis. Please clarify why wasn't kraken2 used, which Kraken database was used and how this might affect the results? Was it necessary to remove these reads? Was this also done for short-read data?

Although the name could suggest otherwise, Kraken2 should not be seen as an improved version of Kraken. At its core, Kraken2 works with different data structures (bloom filters) that allow the software to run faster and under much lower requirements of memory. However, this data structure compromises its overall accuracy, and in fact, we have observed Kraken to have a better accuracy than Kraken2 to classify MTBC reads. Additionally, we use Kraken with an ad-hoc database that we have previously comprehensively benchmarked and, in particular, we have shown to work well for the specific task of removing non-MTBC reads (Goig et al., BMC Biology 2020). In the same work, we show why it is necessary (or at least a good practice) to remove these reads, not only in MTB studies, as non-MTBC reads can also map to the reference genome and interfere with variant calling. In fact, currently, many genomic studies of MTB have incorporated this as a basic QC step. This has been tested with short reads; thus, we systematically applied this filter when analysing short-read data. This specific detail has now been explicitly included in the Methods section (lines 193-194).

R2.5. Why did the authors opted for a strict molecular clock in BEAST when the rate can vary across branches? Did the authors attempted a different approach using other molecular clock models?

We thank the reviewer for this important point. Our aim here was not to obtain the best possible estimate of the *MTB* molecular clock, but to compare clock estimates across genome representations (short-read mapping excluding repetitive regions vs. long-read assemblies including the complete genome) under a consistent modelling framework.

We opted for a strict molecular clock for two main reasons. First, in *MTB* the substitution rate is low compared to many other pathogens, and in datasets like ours—particularly with a largely homochronous sampling scheme—the temporal signal available for tip-dating is limited; therefore, introducing clock-rate heterogeneity often adds little inferential benefit while substantially increasing model complexity. Second, relaxed uncorrelated clock models introduce many additional parameters, typically requiring substantially longer MCMC runs to achieve adequate mixing and convergence, which can be inefficient when the objective is primarily comparative.

Consistent with our previous dating analyses on MTB datasets (López et al. 2023, Cancino-Muñoz et al. 2022, Saavedra Cervera et al. 2022, López et al. 2020), we have generally observed very similar posterior medians for key dating parameters (including clock rate) under strict versus uncorrelated relaxed clocks, while relaxed-clock models more frequently showed poorer mixing and longer convergence times. Given that our analysis was explicitly comparative (same isolates, same tree-height, same priors and settings across genome representations), we considered the strict clock an appropriate and robust choice to address the study question.

We have included this explanation at the beginning of the specific method section.

Lines 229-231: *Rather than estimating the evolutionary rate of M. tuberculosis per se, this analysis focused on comparing evolutionary rate estimates derived from short-read mapping data, excluding complex, repetitive regions, and from complete genomes, under a consistent modelling framework.*

RESULTS

R2.6. The evolutionary rate observed when considering complete genomes is about 1.5 fold higher. Do the authors credit this to the highly variable regions excluded? When performing the ascertainment bias correction in BEAST did authors used different genome lengths, and hence different number of invariable sites in short read data since regions difficult to map were excluded? I am asking because when using sequences with higher number of variants without correcting for this it might, by itself, lead to higher substitution rate estimates.

We thank the reviewer for raising this question. We applied ascertainment bias correction in BEAST in both analyses (short reads and complete genomes) by specifying the number of invariant sites in the XML. Importantly, we used the effective genome length corresponding to each analysis, so that the count of invariant sites matches the genome portion actually

analyzed. This avoids inflating the substitution rate simply because one dataset contains more observed variants.

We revisited the short-read data configuration and confirmed that the ascertainment-bias correction in the short-read analysis had been parameterized using an unadjusted genome length (i.e., not accounting for the exclusion of difficult-to-map sites). After updating this consistently, the complete-genome vs. short-read rate difference remains essentially unchanged, with the ratio being ~1.44-fold (rather than ~1.5-fold).

To assess whether the higher rate is driven by highly variable regions included in the complete genomes, we performed an additional complete genome analysis in which we masked the evidence of gene conversion in the PE/PPE regions. Under this filtering, the rate difference relative to short reads decreased from ~1.44-fold to ~1.25-fold (see figure below). This suggests that detectable gene conversion events contribute to the accelerated rate in complete genomes, although a substantial fraction of the difference remains, suggesting additional sources of variation also contribute.

We hypothesize that (i) gene conversion may also occur in other repetitive gene families (e.g., ESX), and (ii) intrinsically higher variability within repetitive regions may further contribute to the higher rate observed with complete genomes.

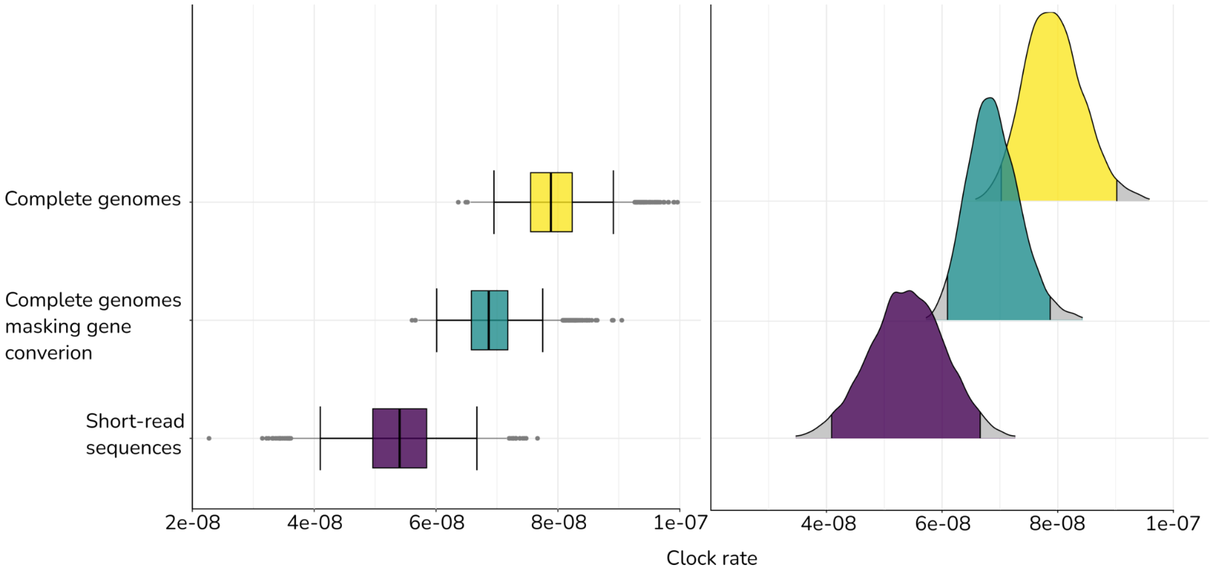

We have included the new results in the manuscript (lines 451-462) and added some lines in the discussion:

Lines 722-728: *Taken together, these observations suggest that the higher evolutionary rate inferred from complete genomes is partly driven by variation within complex, repetitive regions. Masking detectable gene conversion in pe/ppe genes reduces this difference to 1.25-fold, suggesting that this source of diversity strongly contributes to the accelerated evolutionary rate. However, a substantial fraction remains, consistent with additional contributions from undetected gene conversion in other repetitive gene families or from diversity hotspots detected in Fig. 2a.*

R2.7. Line 455 – The authors mention that some regions were completely absent. Could this be confirmed by PCR or other method? To confirm that this is not a sequencing/assembly issue.

While we appreciate the suggestion for further validation, we believe additional confirmation may not be necessary, as the analysis of mapped reads (against the CDC1550 reference genome with the complete locus) from both Illumina and PacBio platforms provides clear evidence of these missing regions.

According to genome assembly data, locus *ppe38-71* was absent in 2 samples (there is a typo in the main text that we have corrected). One of the samples belongs to *Mycobacterium bovis* species (G1706), which is known to lack this locus, therefore acting as a positive control. The other sample belongs to lineage 4.3 and has a large deletion in that region, including the whole locus.

We attach screenshots of the BAM files visualized with Tablet with the two different sequencing technologies (short and long reads) that show the gap against the CDC1550 reference containing the whole locus. Additionally, we included a positive control of one sample from lineage 4.1 to show that the region can be successfully sequenced and analyzed by both sequencing technologies. The black vertical lines delineate the specific region.

Sample G1663 (L4.3). Short-read sequencing

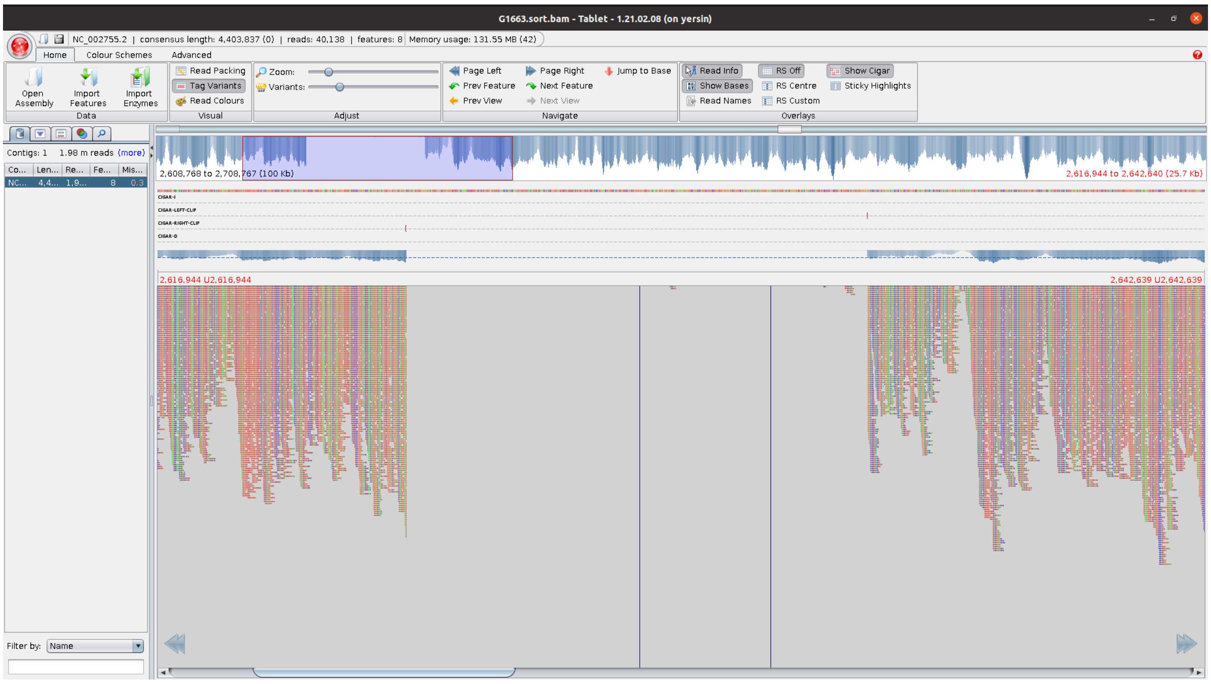

Sample G1663 (L4.3). Long-read sequencing

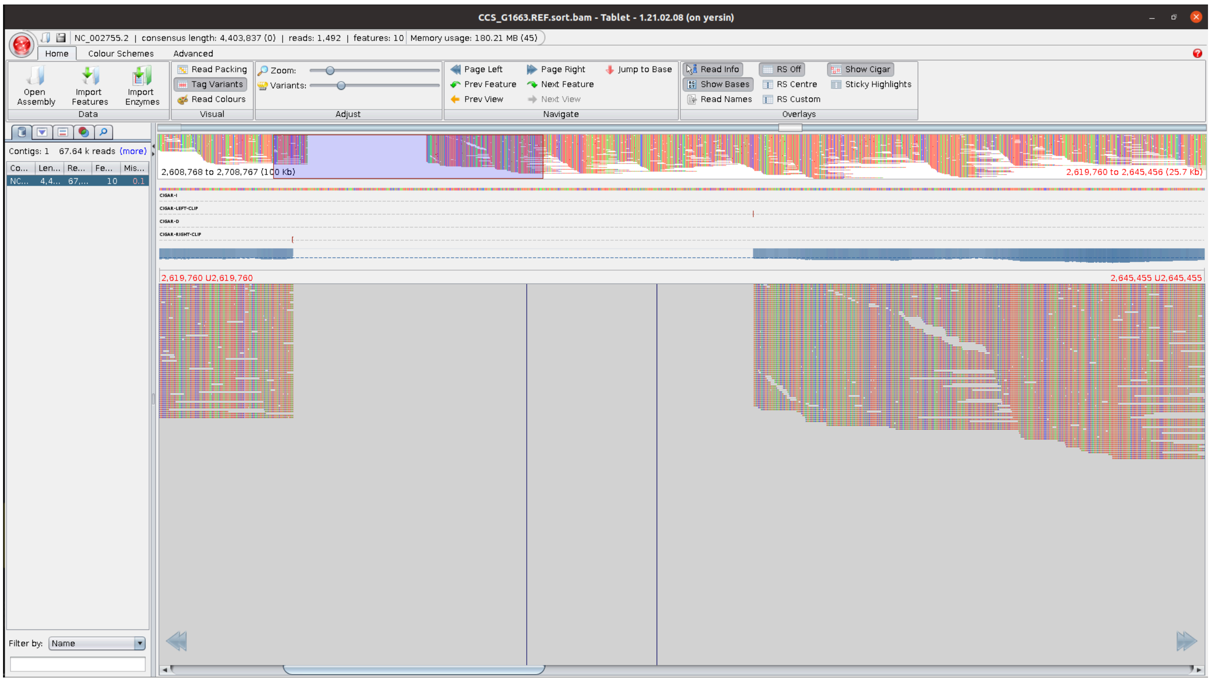

Sample G1706 (*M. bovis*). Short-read sequencing.

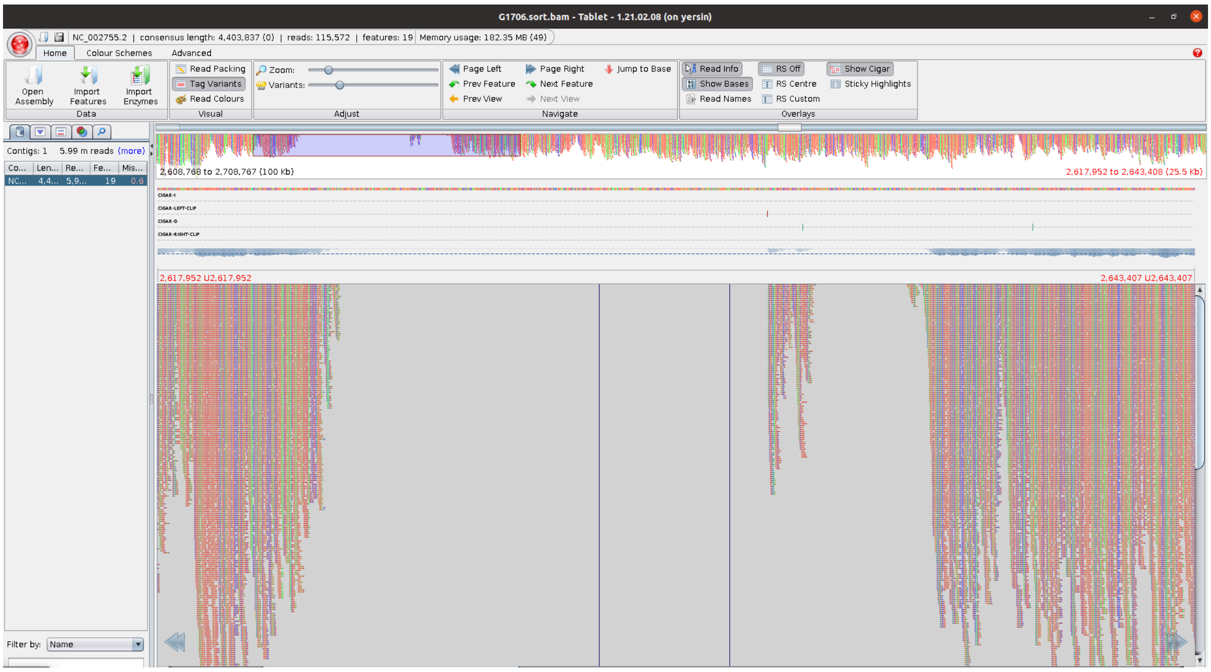

Sample G1706 (*M. bovis*). Long-read sequencing

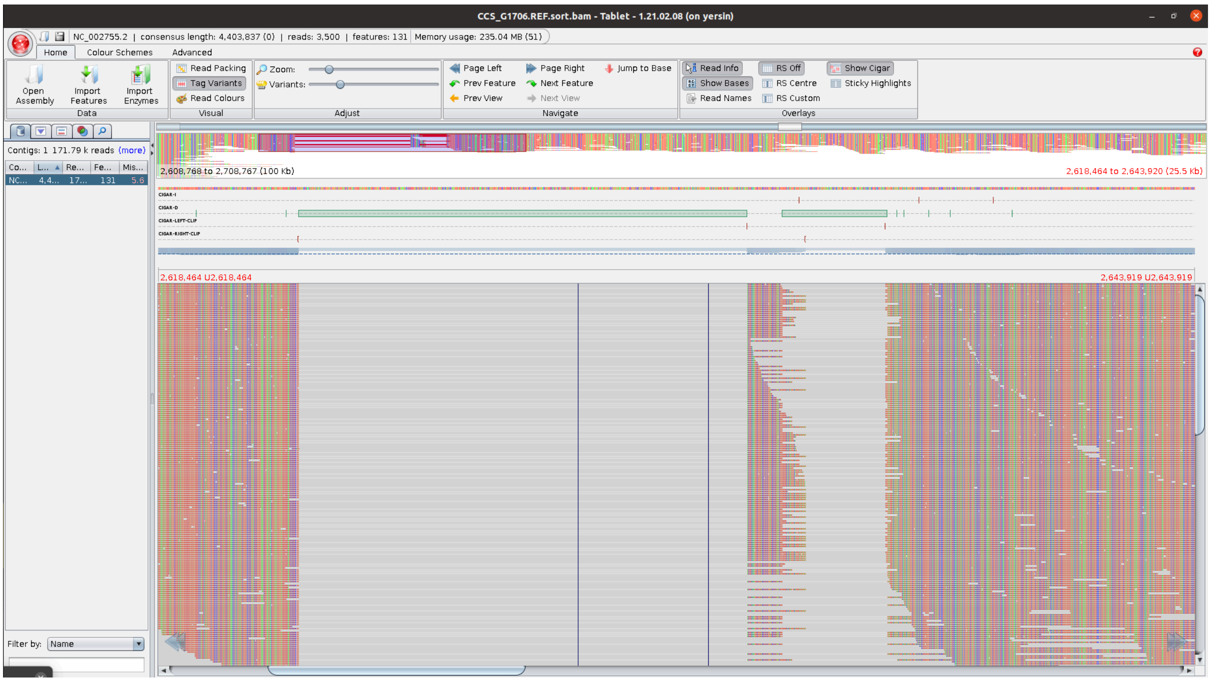

Positive control. Sample G1573 (L4.1). Short-read sequencing

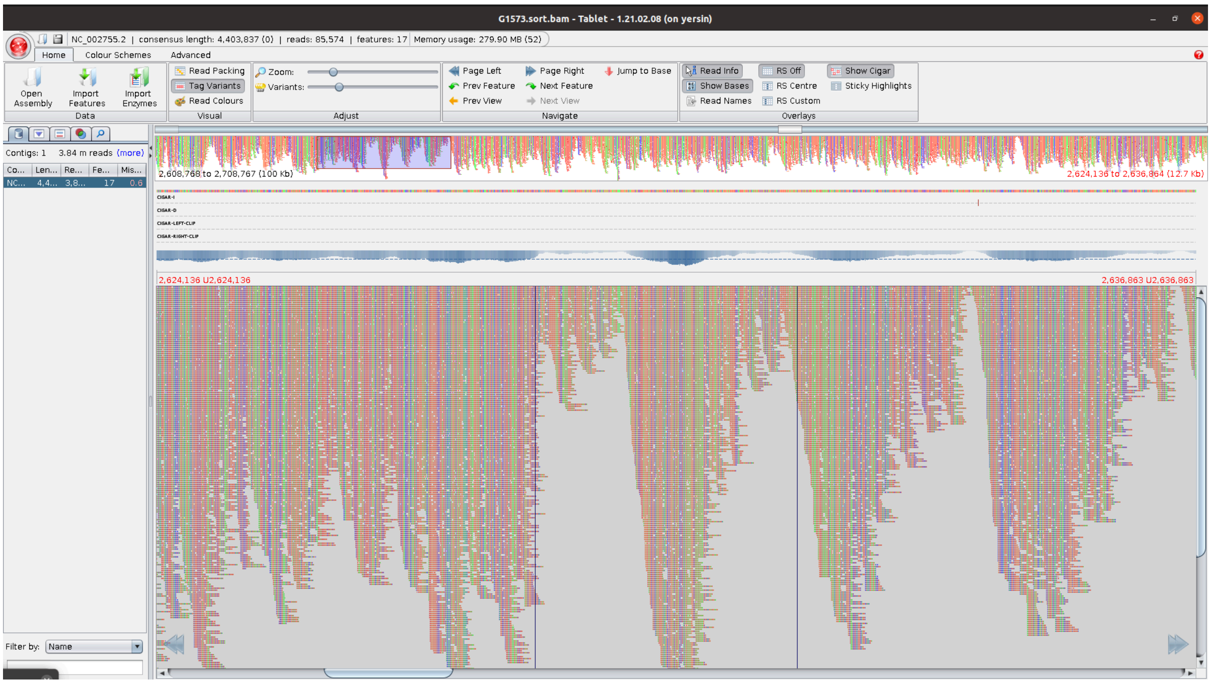

Positive control. Sample G1573 (L4.1). Long-read sequencing

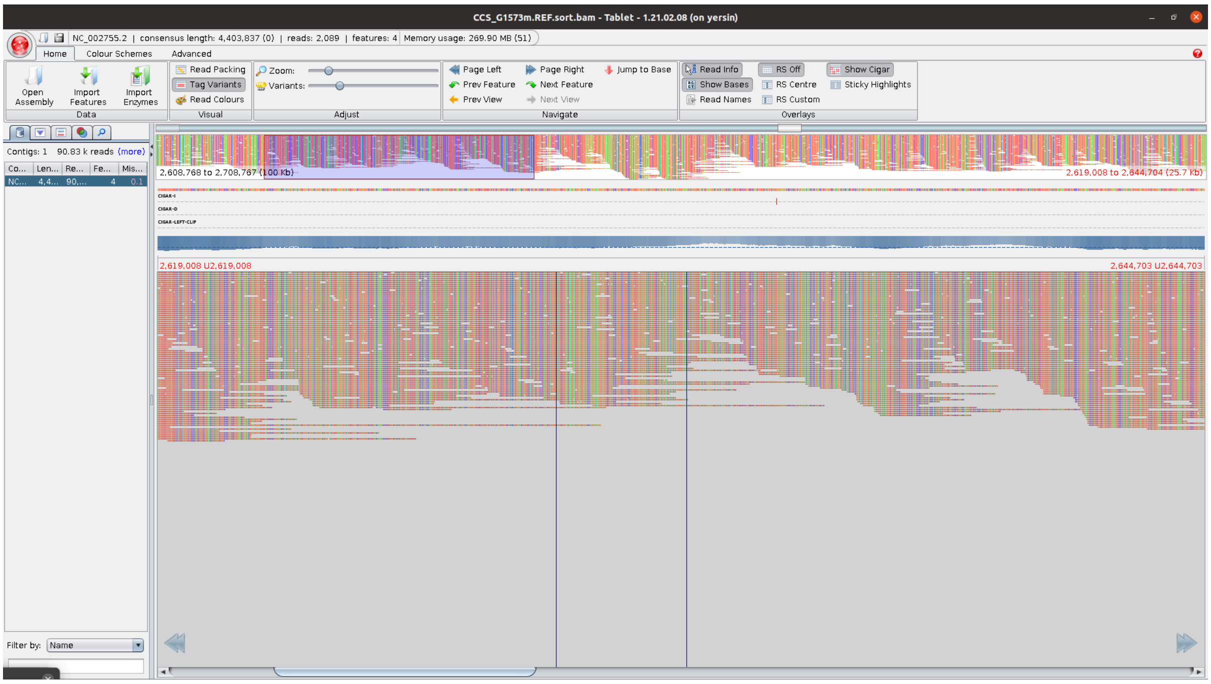

R2.8. Would it be possible for the authors to describe if there is any clear association of gene conversion events and the phylogenetic structure of the tree, i.e., association with specific branches or subtrees and when did these might have occurred? Or, if there is evidence of independent occurrences of similar gene conversion events? There is a tree depicting the distribution, but it is not well addressed in the text.

As suggested by the reviewer, we have now specifically focused on the confirmed gene conversion events detected in *pe/ppe/pgrs* genes and how they are distributed in the phylogeny:

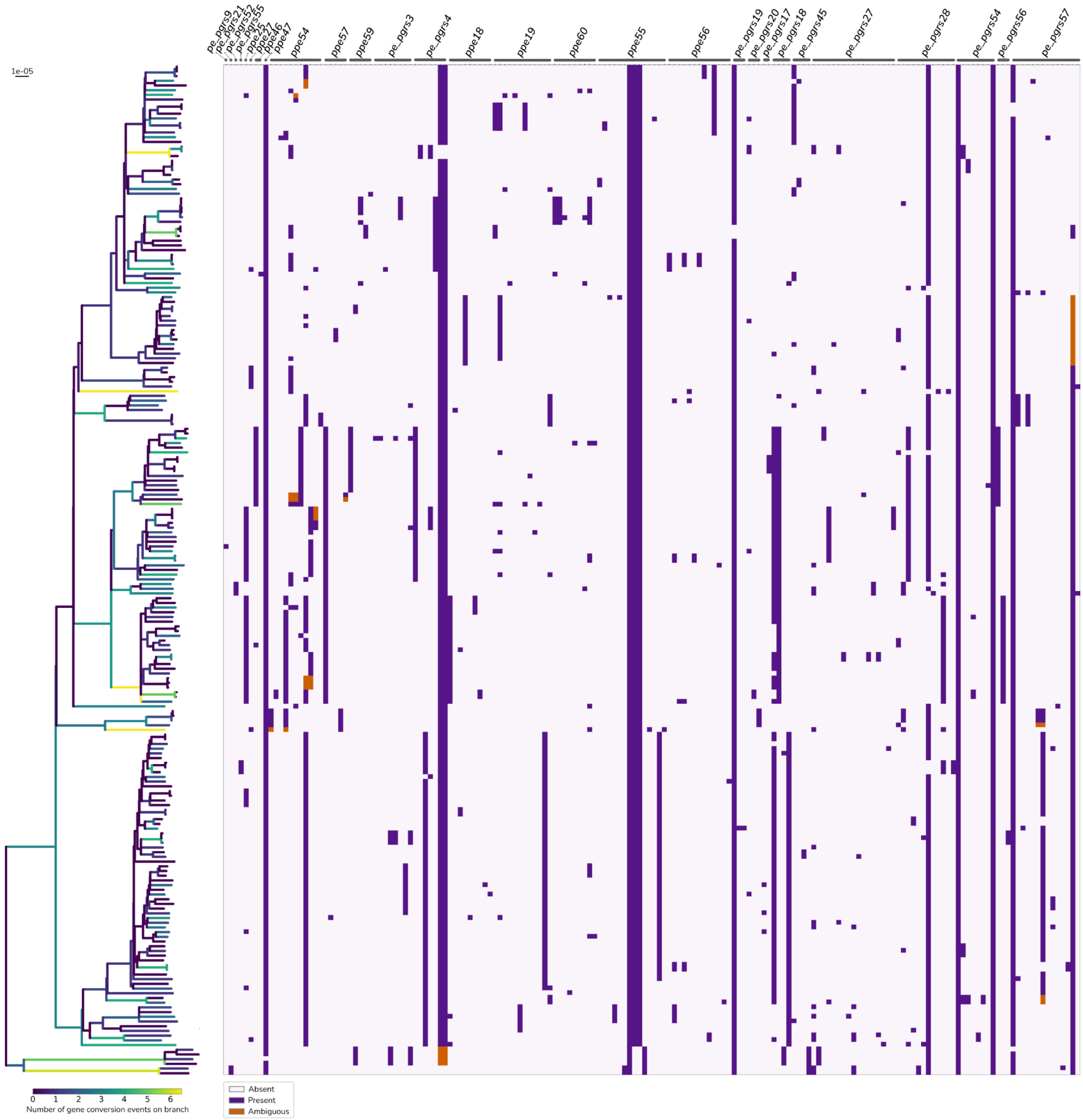

Most of the events appear once, but there are several instances of convergent evolution (see below), as expected when there is gene conversion, including an event happening 13 times in the phylogeny. We have measured this convergent evolution by analyzing the number of parsimony steps over the short-read-based phylogeny:

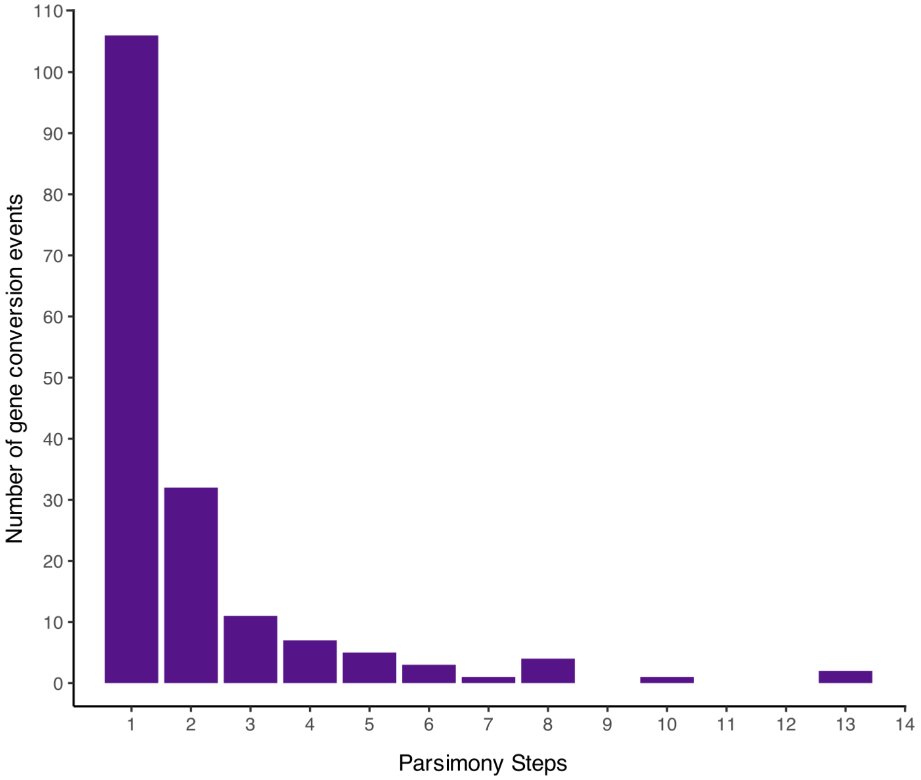

As suggested by the reviewer, we have now also dated the age of each event. For most events, their age coincides with the expansion of TB during the XVIII-XIX centuries.

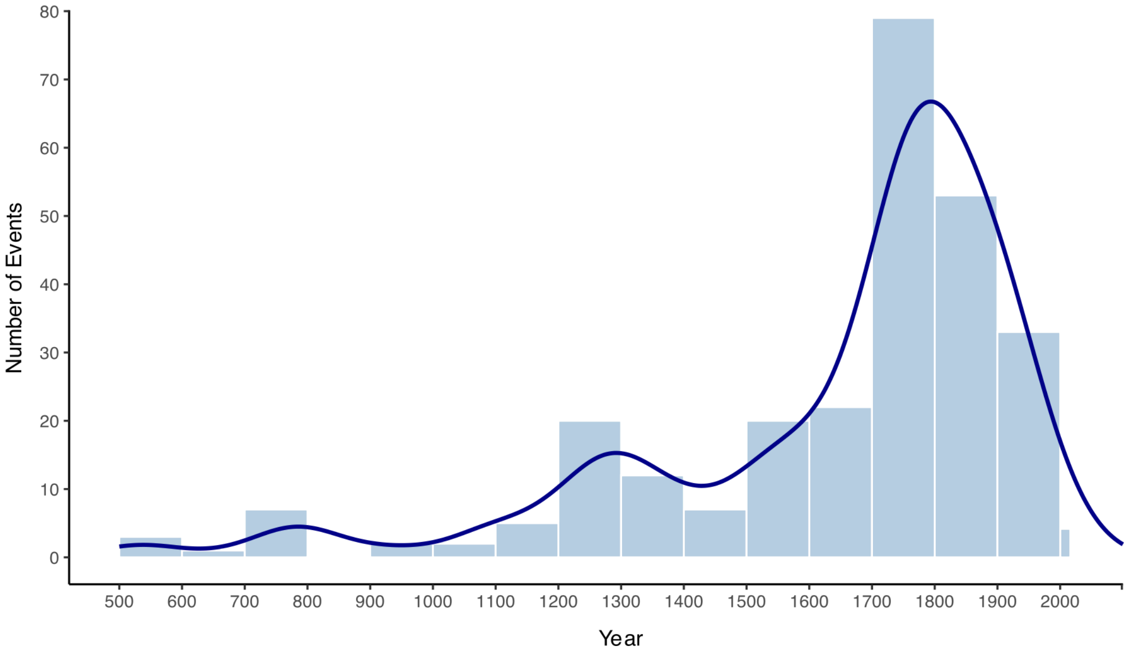

We have replaced in the manuscript the figure obtained using GUBBINs, as it was just a preliminary analysis (as indicated) with the phylogram+heatmap figure. We have included a supplementary table containing all the gene conversion events identified and the isolates where they are present, and the distribution of the parsimony steps of the events and their distribution by year of occurrence as supplementary figures.

These new results are now described in the following paragraph:

Line 521-525: *The distribution of these gene conversion events was closely related to branch lengths (Fig. 3c, left panel) (Spearman's rho = 0.84, p < 0.01). Most events occurred only once, though some mapped to multiple branches of the tree. We quantified this convergent evolution by analyzing the number of parsimony steps on the short-read-based phylogeny (Supplementary Fig. 4a). A majority of these events align with the expansion of TB during the XVIII-XIX centuries (Supplementary Fig. 4b).*

R2.9. Figure 7 – It is interesting to observe that some pairs of isolates distancing 1 SNP when using short-read data end up at 15/16 SNPs apart when using complete genomes. Looking at the increase generally observed for the rest of the data, the magnitude of this increase is unusual. What does account for this, and can the authors offer an explanation?

We agree that these cases are interesting, although they do not represent the general situation. These increases correspond to only two samples:

As indicated in the manuscript, one cluster involved a strain with a gene conversion event, which introduced a large number of mutations (14 SNPs) and generated four dramatic increases in the Shankey plot. While gene conversion is less likely to happen at the epidemiological scale, a similar situation for a single outbreak was described by Stritt et al., 2025. It occasionally can happen at short genetic distances and help to resolve transmission events, as shown in our data in Cluster 10 (mentioned in lines 651-653), where the event was considered as a single mutation in the reconstruction of the genetic transmission network.

The other case involved one sample that harboured several SNPs in different *pe/ppe* genes and other difficult-to-resolve regions, as shown in the following table:

Complete genome ID1	Pos ID1	WT ID1	Complete genome ID2	Position ID2	WT ID2	Reference genome	Position REF	WT REF	Locus	Gene	Featu re	Annotation filter
CCS_G1676	1550002	C	CCS_G1829m3	1550055	G	MTBC ancestor	1561330	G	IG1408_Rv1385-Rv1386	-	IG	DISCARD
CCS_G1676	1562467	A	CCS_G1829m3	1562520	G	MTBC ancestor	1573796	A	Rv1396c	*PE_PGRS25*	CDS	DISCARD
CCS_G1676	1627131	T	CCS_G1829m3	1627184	C	MTBC ancestor	1638211	T	Rv1452c	*PE_PGRS28*	CDS	DISCARD
CCS_G1676	1627132	T	CCS_G1829m3	1627185	C	MTBC ancestor	1638212	T	Rv1452c	*PE_PGRS28*	CDS	DISCARD
CCS_G1676	3215346	A	CCS_G1829m3	3215400	G	MTBC ancestor	-	-	IG2965_Rv2920c-Rv2921c	-	IG	KEEP
CCS_G1676	3215458	G	CCS_G1829m3	3215512	A	MTBC ancestor	-	-	IG2965_Rv2920c-Rv2921c	-	IG	KEEP
CCS_G1676	3215626	G	CCS_G1829m3	3215680	A	MTBC ancestor	-	-	IG2965_Rv2920c-Rv2921c	-	IG	KEEP
CCS_G1676	3750431	G	CCS_G1829m3	3750485	T	MTBC ancestor	3767082	T	Rv3350c	*PPE56*	CDS	DISCARD
CCS_G1676	4344740	T	CCS_G1829m3	4344794	C	MTBC ancestor	4350884	C	Rv3872	*PE35*	CDS	DISCARD

R2.10. Concerning the discussion a popping question is: how does the integration of additional resolution in transmission scenarios help with our understanding of transmission and our capacity to detect and act upon. This is something the authors aim at but a clear vision for this integration or its need is not discussed or put into a broader perspective.

We appreciate the comment, and we have now expanded the discussion around this topic, including the potential public health relevance of using complete genomes either by themselves or for reference-based mapping. Here is the paragraph:

Lines 765-772: *While the increase in resolution is modest, it is important to note that the 0–5 SNP range yields the most actionable results for public health intervention. Consequently, a variation of even one or two SNPs can be decisive in determining whether an investigation is initiated. Furthermore, our analysis can provide stronger support for those cases with 0 SNP differences, improving the inferences derived from short-read approaches. Although the limited number of confirmed contact pairs in our current dataset precludes definitive conclusions, larger benchmarking datasets spanning a broader timeframe will help formally test this trend. While genetic network analysis is not definitive, improved resolution provides valuable insights for outbreak investigations, better guiding the allocation of public health resources.*

Isolate_ID	Isolate_ID	Detected by public health	Days	Illumina mapping	Complete	
G1498	G1499	yes	36	634	928	
G1544	G1630	yes	2	0	0	
G1693	G1661	yes	23	3	3	
G1653	G1785	yes	47	0	0	
G1537	G1786	yes	109	1	0	*False positive SNP in Illumina data
G1616	G1967	yes	29	721	1066	
G1604	G1704	yes	111	0	0	
G1704	G1821	yes	66	1	2	
G1604	G1821	yes	177	1	2	

Minor Issues:

R2.11. Line 75-77 – at this stage, this sentence may be confusing as no background was given before.

Thanks for the suggestion. We agree with the reviewer, and we have added a paragraph to introduce the limitations and relevance of within-host diversity (lines 53-61) as indicated in R2.2

R2.12. Lines 186 – The authors mention the WHO mutation catalogue as a basis for identifying resistance associated variants. Yet some mutations, owing to its low frequency or association with specific clades, may still be classified as mutations with uncertain association. How was this dealt with?

As the reviewer points out, drug resistance profiles were predicted based on the identification of resistance-associated mutations in the WHO catalogue, together with the additional expert rules recommended in the catalogue. The MTBC isolates included in this study were previously analysed in a larger short-read sequencing study assessing the accuracy of genomic drug resistance prediction (García-Marín et al. 2024). Using long-read data, we recovered the same resistance profiles. In line with the results of the previous study, only isolates carrying the *inhA* S94A mutation—which had an uncertain association with resistance in the first version of the catalogue—were classified as resistant. We have added a clarifying sentence in the manuscript to make this explicit.

Lines 201-204: *Drug resistance profiles were determined by identifying mutations associated with resistance, as listed in the first version of the World Health Organization (WHO) catalogue, and considering the expert rules. In line with a previous study assessing genomic resistance prediction in our setting (García-Marín et al. 2024), isolates carrying the inhA S94A mutation were classified as resistant.*

R2.13. Lines 272-275 – did any type of epitope sequence clustering was done at IEDB to remove redundancy between epitope records. It is possible that there is overlap between sequence entries that may relate to the same epitope.

Epitope sequences were retrieved directly from IEDB, without any prior clustering to remove redundancy. Any overlap between epitope records was handled during downstream processing: epitope sequences mapping to overlapping genomic regions were merged into a single non-overlapping sequence, so that each region was represented only once in the analysis. This step is now described in more detail in the revised Methods:

Lines 330-332: *Of those, 124 overlapping epitope sequences were merged into a single large sequence to generate non-overlapping epitope sequences. Finally, we generated 115 non-overlapping epitope sequences belonging to 61 pe/ppe genes.*

R2.14. Line 285 – Please clarify how this dN/dS ratio was obtained. Is it the medians from pairwise comparisons in an alignment? How did you ensure a codon-aware alignment at this scale of genomes and genes?

As indicated in lines 284–287 (old manuscript), dN/dS ratios were calculated using SNAP. For each gene, SNAP computes pairwise dN/dS values between the MTBC ancestor reference genome included in our MSA and each corresponding gene sequence. The median dN/dS value across these pairwise comparisons was then used as the summary statistic.

The generation of the multiple sequence alignments is described in lines 252-256 (old manuscript). The MSA for each *pe/ppe* gene was generated using MACSE, a codon-aware aligner that explicitly accounts for reading frames and frameshifts. This ensured that alignments were maintained at the codon level across all genes and genomes. In addition, all alignments were visually inspected using AliView to identify and correct potential alignment artefacts. The alignments of epitope and non-epitope regions were extracted from the corresponding MSA by gene coordinates, and then merged into concatenated alignments (otherwise, the low genetic diversity restricts the measure of the dN/dS ratio in some cases).

We have modified the paragraph to clarify both questions:

Lines 333-343: *We adopted the methodology described by Comas et al. 2010 to assess evolutionary signatures in pe/ppe genes. Briefly, sequences were classified into three categories: epitope regions, non-epitope regions, and complete antigen sequences. For each category, MSAs were generated by extracting the corresponding regions from the pe/ppe MSAs described above and concatenating them using FastaCon. The number of synonymous and non-synonymous substitutions was then estimated using SNAP (Rodrigo and Learn 2007), which implements the Nei and Gojobori method (Nei and Gojobori 1986). For each concatenated alignment, individual sequences were compared pairwise against the MTBCA reference genome to obtain dN/dS values. Median dN/dS ratios were subsequently calculated across all pairwise comparisons for each category and compared using the Wilcoxon signed-rank test. In addition, the same analyses were repeated for epitope, non-epitope, and complete antigen sequences after masking gene conversion events (Didelot and Parkhill 2022).*

R2.15. Figure 2- Please explain better in the legend that the distribution by COGs is just for the genes associated with mutation hotspots, correct?

We thank the reviewer for suggesting this clarification. We have added the following line to the manuscript:

Line 506-507: *Distribution of COG categories of genes with the highest levels of segregating sites.*

Reviewer #1 (Remarks to the Author):

Key results
This resubmission presents a large set of PacBio HiFi complete genomes from clinical isolates in Valencia and uses those assemblies to argue that complete genomes recover substantial additional variation beyond standard short-read analyses, identify pe/ppe diversity hotspots shaped by gene conversion, improve transmission resolution, and revise current views of within-host diversity. The dataset is potentially valuable. However, after revision, I remain unconvinced that the manuscript supports its current title, abstract, and central framing. The paper still presents a largely Lineage-4 regional dataset as a general map of Mycobacterium tuberculosis genetic diversity across evolutionary scales. That mismatch between dataset and claim remains the principal obstacle to publication.

Validity
1. The main scope problem remains unresolved. The manuscript still makes species-level claims about Mycobacterium tuberculosis genetic diversity, yet the study population is a regional Valencia cohort overwhelmingly dominated by Lineage 4. Adding statements that the dataset is “mostly” or “enriched in” Lineage 4 does not solve the problem when the title, abstract, and conclusions continue to generalize to M. tuberculosis as a whole.

We respectfully disagree, as there is no indication that the rest of the lineages do not follow similar diversity generating mechanisms. Genome-wide diversity, PE/PPE diversity, and gene conversion, epitope conservation, all these patterns are expected to hold for the whole MTBC as they all share the same evolutionary mechanism and are 99.9% similar among them. This has been shown repeatedly in the analysis of large collections of short-read data. In addition, our dataset, while coming from one geographic setting, is derived from both local and imported TB cases, so many isolates are not from local diversity (35% foreign-born individuals from 23 countries). Analysis of imported TB cases has been used historically to understand the global diversity of Mtb.

2. The within-host conclusions remain broader than the data support. This part of the study analyzes serial short-read isolates from only five patients, mapped to patient-specific baseline complete genomes. That design can support a narrower conclusion about the effect of reference choice on false-positive non-fixed variant calls, but it does not justify a broad reassessment of within-host diversity in M. tuberculosis.

We appreciate the reviewer’s caution regarding the scope of our conclusions. We would like to draw his/her attention to the fact that our conclusions are supported by two complementary datasets: (i) Five patients with serial isolates, which allowed us to use patient-specific complete genomes as reference, (ii) 372 *M. tuberculosis* isolates from 77 genomic transmission clusters, which allowed us to use cluster-specific complete genomes as reference. By combining these datasets, we observed a high proportion of false-positive non-fixed SNPs when mapping against a standard global reference. Because non-fixed variants are critical for understanding drug resistance, polyclonal infections, and transmission, the high rate of false positives we identified suggests that current methods may lead to inaccurate interpretations. Therefore, we do not present a "new theory" of diversity, but rather suggest that a reassessment of how these variants are called is necessary to ensure the accuracy of future clinical and epidemiological studies.

3. The structural-variation component is still not presented as a systematic genome-wide analysis. Selected findings at ppe38-71 and limited epitope-focused observations may be interesting, but they do not support broad statements about the structural-variation landscape or its overall contribution to diversity.

The broad statement suggesting systematic structural variation analysis across the dataset has been removed in the latest version to prevent misunderstanding. We have checked for any remaining ambiguity on this topic, ensuring the manuscript's scope is clear. Structural variation

analysis was performed in the specific context of *ppe38-71*, epitope regions within PE/PPE antigens, and the genomic epidemiology section.

4. The gene-conversion section is improved relative to the original submission and is no longer my main concern. The revised methods are clearer and the orthogonal validation is a genuine improvement. Even so, that technical progress does not overcome the central framing problem described above.

We appreciate the reviewer's positive comments regarding the improvements to the gene conversion section and the updated methodology. Regarding the manuscript's framing, we believe the revisions made in the previous round, specifically the adjustments to the Introduction and the narrowing of our claims, addressed these concerns. In the last version, we carefully revised the text to ensure the study's focus was better defined and that our conclusions were more closely aligned with the results presented. We believe that, with the technical concerns now resolved, the current structure of the paper clearly supports our findings.

Significance
Because the dataset is overwhelmingly Lineage 4 from one geographic setting, the current manuscript does not support the breadth of significance claimed for a general-interest journal audience. The work would be more compelling and more credible if it were framed as a focused study of a Lineage-4-enriched population-based complete-genome dataset and its implications for repetitive-region diversity, reference choice, and selected local epidemiologic inferences. In its current form, the manuscript overstates the generality of the conclusions.

See above responses.

Data and methodology
5. The complete-genome dataset itself appears valuable and the manuscript is stronger than the original version in describing how gene-conversion candidates were identified and cross-checked.

We appreciate the reviewer's positive feedback on the updated manuscript. We are glad that the addition of orthogonal methods to identify gene conversion candidates has addressed the previous concerns and added more rigor to our findings.

6. However, the annotation strategy remains limited for discovery of lineage- or isolate-specific gene content. A lift-over strategy anchored to H37Rv is practical, but it does not provide a true pangenomic view and therefore cannot support broad conclusions about novel gene content or gene-content evolution.

Since the beginning, we have never used the term pangenome and we have not made any statement about novel gene content or gene-content evolution in our manuscript, precisely because we don't carry out de novo annotations. It was not the scope of the manuscript, as it is a whole different topic and a separate piece of work.

7. For structural variation, the current analyses remain selective rather than comprehensive. If the authors wish to retain stronger SV claims, they need a systematic genome-wide calling framework and a quantitative summary of events, sizes, frequencies, and affected loci.

While we agree that a de novo annotation approach and a systematic, genome-wide structural variation (SV) framework would provide a more exhaustive view, we feel these analyses fall outside the intended scope of this paper. Expanding the study to include a full pangenomic reconstruction would be a massive undertaking, likely enough to constitute a separate study entirely, and we are concerned that it would dilute the primary focus of this manuscript.

To address the reviewer's concerns in the first revision, we have removed the specific phrasing in the introduction that previously led to misunderstandings regarding the scope of our SV analysis, and we have ensured the manuscript no longer makes broad, generalized claims about genomic diversity driven by SVs. We have restricted our SV-related conclusions to the specific sections where those analyses were actually applied, specifically within the epitope and epidemiological analyses. We believe this more focused approach ensures our conclusions are fully supported by the data presented without overreaching into a comprehensive SV survey.

Analytical approach
8. Key quantitative analyses are effectively Lineage-4 analyses. That is acceptable for the dataset at hand, but it reinforces the need to narrow the manuscript-wide claims.

We would like to clarify that only one specific quantitative analysis is focused on Lineage 4. This was due to a limitation in measuring the evolutionary rate because the samples are all from the same year, rather than a limitation of the enrichment in L4. Importantly, our purpose was to accurately estimate the contribution of previously masked regions to the overall clock rate. That contribution is not expected to differ to a large extent by lineage, given that similar mechanisms and population processes act in all of them. As we've noted in our previous responses, we have already adjusted the text to ensure our broader claims are properly contextualized and supported by the data.
9. The comparison between universal-reference and patient-specific-reference pipelines is informative, but the interpretation should be restricted to what was actually tested: false-positive behavior in short-read calling, not the full extent of within-host diversity accessible with complete genomes.

This is acknowledged in comment 2 and the previous revision.

10. The revised analyses are technically improved, but the inferential reach of the manuscript still exceeds the reach of the underlying study design.

See above comments

Clarity and context
The manuscript is clearer than the previous version, but the central message remains internally inconsistent: the authors acknowledge the Lineage-4 enrichment and then continue to frame the study in organism-wide terms. That tension should be resolved directly. There are also residual textual issues that suggest the paper still needs careful editorial polishing, including duplicated phrasing and a few wording or figure-reference problems.

Thanks for pointing out, we have now gone through the manuscript and tried to correct any incoherence.

References
The literature context is improved, but some of the broad interpretive statements still outpace the evidence presented. The manuscript would benefit from more explicit positioning relative to prior work on Lineage 4, MTBC pangenome diversity, and structural variation.

See above comments.

Reviewer #1 (Remarks on code availability):
My assessment covers bacterial genomics, comparative genomics, tuberculosis genomics, variant calling, and evolutionary interpretation. I did not independently verify every computational intermediate, but the main concerns raised above are conceptual and inferential rather than dependent on re-running the full pipeline.

Reviewer #2 (Remarks to the Author):

This revised version incorporates several changes that have clarified some aspects that were not entirely clear. Furthermore, the authors answered all my questions and addressed the concerns that I have raised by undertaking further data analysis and integrating the results in the main text for discussion and analysis. I have some additional and minor comments:

As an optional point for discussion - the authors determined an increased mutation rate upon including full genome sequence data. Should this type of sequence data be analysed at the light of less stringent cutoffs?

While different cutoffs for genome assembly can be used, we think that our current cutoffs are the most appropriate to avoid inclusion of false positives that may artificially increase clock rates.

In the attempt to respond to the concerns raised by Reviewer 1, the authors have changed the title to include the term "evolutionary scales" to characterize the scope of the sample and the breadth of conclusions that can be drawn. Personally, I find this term poorly informative and quite vague. I do agree that the overall genetic diversity of this sample is somewhat limited, but this can be discussed and acknowledged as a limitation.

Thanks for the suggestions. We believe across evolutionary scales reflects the fact that we provide data at the population, epidemiological, and within-host scales, and this should be reflected in the title.

L301-304 – The authors refer to annotation by lift over "as previously described" yet provide no reference.

We referred to a previous method section. Sorry for the misunderstanding. We have clarified it in the text.
